# Documentation of vaccine wastage in two different geographic contexts under the universal immunization program in India

**DOI:** 10.1186/s12889-020-08637-1

**Published:** 2020-04-25

**Authors:** Manoja Kumar Das, Mangla Sood, Muralidhar Parashuram Tambe, Thakur Dutt Sharma, Malangori Abdul Gani Parande, Jitendra Bhaskar Surwade, Nandakumar Manikrao Salunkhe, Shital Somsing Patil, Bhagwan Pawar, Rajesh Guleri, Chitra Kaushal, Monica Sindhu

**Affiliations:** 1 The INCLEN Trust International, F1/5, Okhla Industrial Area, Phase 1, New Delhi, 110020 India; 2 Indira Gandhi Medical College, Shimla, Himachal Pradesh India; 3 Department of Health and Family Welfare, Government of Himachal Pradesh, Shimla, Himachal Pradesh India; 4 B.J. Government Medical College, Pune, Maharashtra India; 5 Department of Health and Family Welfare, Government of Maharashtra, Pune, Maharashtra India; 6 Department of Health and Family Welfare, Government of Himachal Pradesh, Kangra, Himachal Pradesh India

**Keywords:** Rotavirus vaccine, Pneumococcal vaccine, Routine immunization, Outreach, Vial size, Vaccine wastage

## Abstract

**Background:**

Government of India is introducing new and relatively costly vaccines under immunization program. Monitoring of vaccine wastage is needed to guide the program implementation and forecasting. Under pilot introduction of rotavirus vaccine in two districts both 5- and 10-doses vials were used, which was considered as an opportunity for documenting the wastage. The wastage rates for other routine vaccines were also documented.

**Methods:**

A survey conducted in two districts (Kangra, Himachal Pradesh and Pune, Maharashtra) covered 49 vaccine stores, 34 sub-centres and 34 outreach sessions collected vaccine receipt, distribution and usage data for two complete years 2016 and 2017.

**Results:**

The overall wastage rates for almost all vaccines were higher in Kangra district (BCG 37.1%, DPT 32.1%, Measles 32.2%, OPV 50.8%, TT 34.1% and pentavalent 18.4%) than Pune district (BCG 35.1%, DPT 25.4%, Measles 21.7%, OPV 14.3%, TT 23.1% and pentavalent 13.2%). Wastage for pneumococcal conjugate and measles-rubella vaccines in Kangra district were 27 and 40.5%, respectively. With transition from 5- to 10-doses vials for rotavirus vaccine, wastage at stores levels increased in both Kangra (29 to 33.2%) and Pune (17.8 to 25.7%) districts. With transition from intramuscular to intradermal fractional inactivated polio vaccine, the wastage increased from 36.1 to 54.8% in Kangra and 18.4 to 26.9% in Pune district.

**Conclusions:**

The observed vaccine wastage rates for several vaccines were relatively higher than program assumption for forecasting. The observed variations in the vaccine wastage indicates need for state or region based documentation and monitoring in India for appropriate programmatic action.

## Background

Under the Universal Immunization program (UIP) in India the vaccine portfolio has been expanded from 6 to 13 vaccines now with 10 vaccines [BCG; oral polio vaccine (OPV); inactivated polio vaccine (IPV); diptheria-pertusis-tetanus (DPwT); liquid pentavalent (LPV) combining DPwT, hepatitis B vaccine (HBV) and *Haemophilus influenzae type b* (Hib); tetanus toxoid (TT), measles and rubella (MR)] universally and three vaccines [rotavirus vaccine (RVV), pneumococcal conjugate vaccine (PCV) and Japanese encephalitis (JE)] in select areas. There is also progressive rise in the full immunization coverage, although slower than anticipated. Usually in the program the vaccine requirements are estimated based on the estimated number of beneficiaries, number of doses and the vaccine wastage. Vaccine wastage is an integral part of vaccine logistics. The Government of India have recommended the wastage rate for 50% (BCG), 25% (Measles, Measles and rubella, rotavirus, and Japanese Encephalitis vaccines), and 10% for all other multi-dose vial vaccines including DPT, LPV, IPV, OPV, TT, and HBV [1]. The vaccines are costly commodities and the newer vaccines are even costlier, putting a significant financial burden on the country. An UNICEF study across 5 states of India informed a high vaccine wastage rates for various vaccines ranging from 27 to 61% for various vaccines [2]. The wastage rates varied across the states for different vaccines. World Health organisation (WHO) estimated that about 50% of the vaccines produced globally are wasted [3]. According to the available reports for PCV pipeline planning in 2010, WHO had analysable vaccine wastage data for only 19 of the 72 Gavi, the Vaccine Alliance eligible countries [4]. Hence it was recommended to reinforce the vaccine wastage monitoring and documentation at national and sub-national levels for large countries like India, with new costlier vaccines being introduced in the UIP. The increase in cost of vaccines makes the solicitous use of vaccines and interest to minimize the vaccine wastage has risen. To reduce the wastage, India adopted open vial policy for liquid vaccines in 2011. Several factors influence the wastage including the vaccine vial, applicability of open vial policy, beneficiary base, session planning, syringes used, immunisation practices, storage and logistics, and national policies [3].

National Technical Advisory Group on Immunization (NTAGI), the apex advisory body for immunization in India recommended phased introduction of RVV in India. NTAGI also recommended a pilot rollout linked to surveillance of adverse events under monitoring of the relevant Government agencies. Pilot introduction of RVV was done in two districts (Kangra, Himachal Pradesh and Pune, Maharashtra) of India, prior to the national introduction linked to surveillance of intussusception, a rare adverse event. Under the pilot introduction, initially five doses RVV vials were supplied, which were changed to 10 doses vials synchronising with the national rollout. The PCV was introduced in Himachal Pradesh (May 2017) under first phase of national rollout. The state UIP program also requested to capture the wastage rates for the other routine vaccines simultaneously. This provided an opportunity to document the vaccine wastage for RVV (two different vial sizes) in two different geographic contexts and PCV under the UIP.

## Methods

### Background setting

Before national introduction of RVV in India in April 2016, a pilot introduction linked to surveillance of intussusception was planned. This pilot introduction was led by Department of Biotechnology in partnership with Ministry of Health and Family Welfare (MoHFW), monitored through an Interministerial-Interagency Coordination Group and funded by Bill and Melinda Gates Foundation. The RVV pilot introduction was launched in two districts Kangra (Himachal Pradesh) and Pune (Maharashtra) in December 2015, which preceded the national introduction (April 2016). The district Kangra continued to receive the RVV supply from the project for 2 years (till December 2017), although the RVV was launched in the state through national rollout in April 2016. The RVV was launched in Maharashtra state (and Pune district) through national rollout in July 2019. District Kangra with population 1.5 million had one district hospital (DH), one sub-district hospital (SDH), 13 community health centres (CHCs), 84 primary health centres (PHCs) and 440 sub-centers. In the Kangra district about 262 fixed sessions and 903 outreach sessions were organised every month. In Pune district, a selected part of the district covering 1 million population with a network of 29 PHCs and six rural hospitals (RH) was included. The immunization and vaccine logistics related health functionaries in these two districts and community mobilisers were trained for implementation through a cascade training system. The RVV was launched in December 2015 and routinely administered to the infants from January 2016. The birth cohorts targeted in these two districts were 41,400 (Kangra 23,400 and Pune 18,000). Three doses of ROTAVAC® (Bharat Biotech) were given at 6, 10 and 14 weeks to the infants. Five doses vials of RVV were used till September 2016 and then 10 doses vials were used, similar to the national RVV supply guideline. The PCV was launched in Himachal Pradesh in May 2017 as part of the national phased introduction with two primary and one booster schedule. The PCV was administered to infants routinely under UIP from July 2017. IPV introduced under UIP underwent transition from 0.5 ml intramuscular to 0.1 ml intradermal (fractional IPV- fIPV) since January 2017. Availability of all these combinations in vaccine vial size, administration route across two different geographies (Kangra- hilly and Pune- plain regions) provided an opportunity to document the vaccine wastages. The state governments also requested to estimate the wastage of the other vaccines under UIP in the districts to inform the program.

The vaccine is supplied from district store to the sub-district stores, cold chain points (CCPs) at CHCs and PHCs on monthly basis. From these CHCs/PHCs, for the sessions at sub-centres and outreach sites, vaccines are supplied using vaccine carriers on the day of session. The unopened and partially used vaccine vials under open vial policy are returned to the store after the session, which are reissued for the next sessions. These vaccine transactions are recorded in the vaccine stock and issue/return register and the children vaccinated numbers are written in the session registers. But the number of remaining doses in the partially used open vial is not written in any register.

### Study design

This cross-sectional study was undertaken in two districts, Kangra (Himachal Pradesh) and Pune (Maharashtra). In each district, the facilities (all levels) providing vaccination and session sites (including outreach sessions) in both urban and rural areas were included to obtain a comprehensive picture of vaccine utilisation and wastage. In Kangra, the DH, 14 CHCS, 14 PHCs, 14 sub-centres were included. In Pune, the DH, five RHs, 4 CHCs, 10 PHCs and 20 sub-centres were included.

### Data collection

These selected 49 facilities (vaccine stores and also service delivery point), 34 sub-centres (service delivery point) and 34 outreach session sites (one per sub-centre) were visited by the trained research staff for data collection. The vaccine supply, logistics and administration data for all UIP vaccines were collected for the period January 2016 to December 2017. Month and vaccine wise data were collected from the multiple registers (at vaccine stores: vaccine stock register- issue and return, vaccine distribution register for sessions and monthly report; at session sites: vaccinator’s logistics diary and RCH register, session and monthly vaccination report) including sessions held, vaccines supplied and returned, vaccine utilisation, children vaccinated, and vaccines discarded or wasted using structured tools (Supplementary files 1 and 2). The interviews with cold chain handlers (CCHs) (*n* = 43), vaccinators (*n* = 31), store-in-charges (*n* = 23), medical officers (MOs) (*n* = 35) and district immunization officers (DIOs) (*n* = 2) were done to gain additional understanding the perceptions, practices and experience related to vaccine usage and wastage handling using semi-structured guide after obtaining informed consent (Supplementary file 3). Double data entry of the data collected was done using a MySQL and PHP based online data entry program followed by matching and corrections. The data was extracted into Microsoft excel format.

### Data analysis

The descriptive analysis performed using Microsoft Excel 2013 and Stata 15.0. The wastage rate at store level and session site levels were estimated using the formula; Wastage rate = Doses wasted × 100 /Doses issued, expressed as percentage. For the stores the doses wasted for specific periods were estimated using the opening stock balance, new doses received, closing stock, and the children vaccinated. For the stores, all doses administrated at the sessions held at facility and outreach sites under the respective store were considered. For the outreach sessions, all doses administrated at the outreach session were considered. Wastage for each vaccine for every month for each facility, session site and store and for each different dose type (IPV and fIPV), vial size (5, 10, 20 and 50 doses), vaccine type (lyophilized and liquid), and applicability of open vial policy (open vial or single use) were calculated. The vaccines under open vial policy in India include OPV, IPV, DPT, HBV, LPV, TT, and PCV, which once opened can be used at more than one session within 28 days, if stored appropriately, handled aseptically and expiry date and VVM are valid [5]. As there was no entry of the returned doses in the partially used open vials from the session sites, we could not estimate the wastage for the open vials at outreach sessions. The reference periods for vaccine wastage rates estimation for Kangra district were January 2016–December 2017 for BCG, OPV, DPT, HBV, LPV, TT, TT, Measles and RVV; April 2016 to December 2016 for IPV; January 2017 to December 2017 for fIPV; July 2017 to December 2017 for PCV and August 2017 to December 2017 for MR vaccine. All the reference periods for the vaccine wastage rates for Pune district were same except the MR and PCV vaccines, as these were not introduced during the observation period. The beneficiary turnout rate (%) was estimated by vaccine doses administered/ number of beneficiaries due on the scheduled day.

## Results

From these selected sites, data for 5276 sessions (Kangra- 3044 and Pune- 2232 sessions) at facilities and 2502 outreach sessions (Kangra- 2166 and Pune- 336) were recorded. A total of 1,004,695 vaccine doses (Kangra- 756,575 and Pune- 248,120 doses) at facility sessions and 159,243 vaccine doses (Kangra- 128,514 and Pune- 30,729 doses) at outreach sessions were administered.

### Frequency of doses administered during the vaccination sessions

**Fig. 1:**
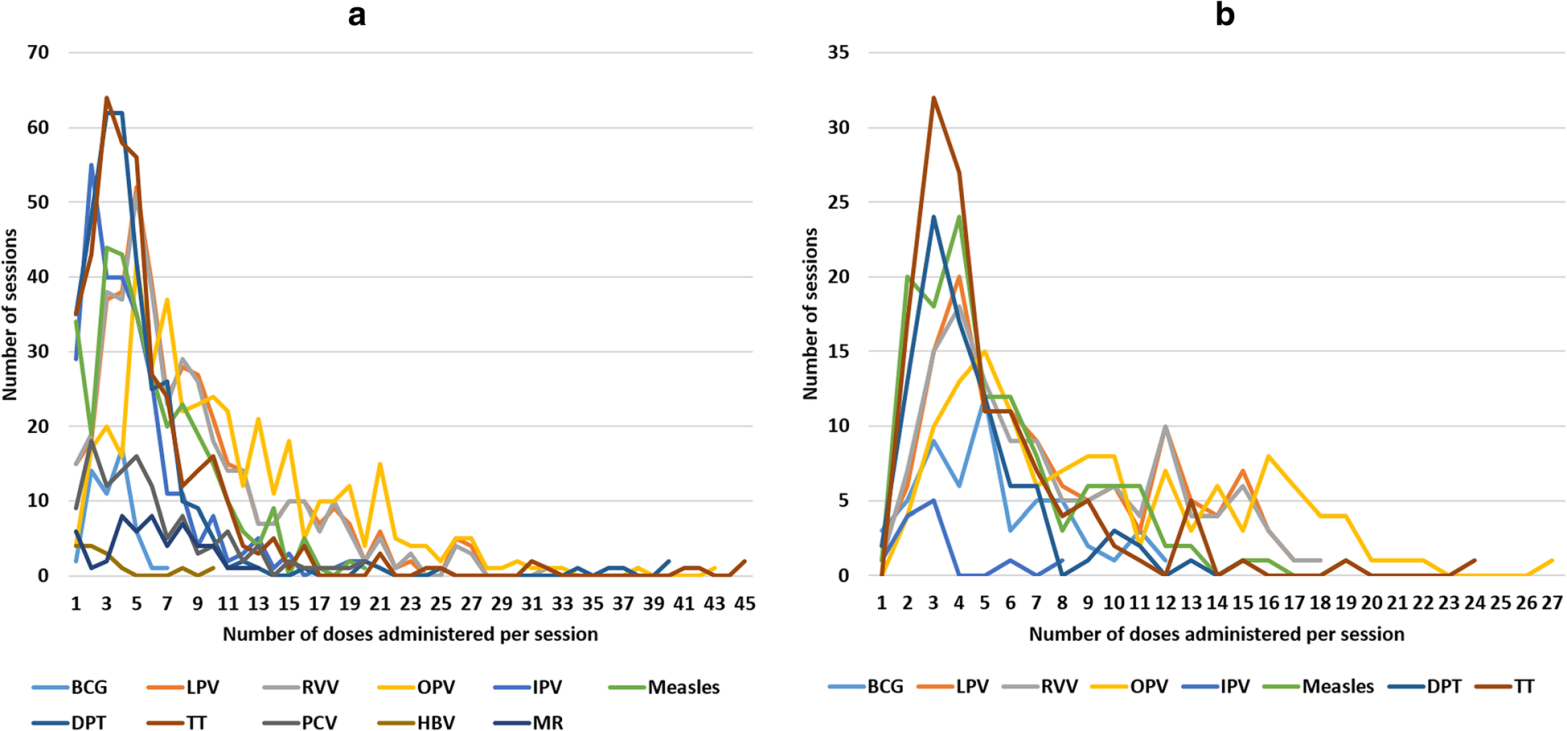
Number of vaccination sessions by the number of doses administered per session. **a** Vaccine doses administered during sessions in Kangra district. **b** Vaccine doses administered during sessions in Pune district

According to the records, the number of doses administered per vaccination session varied widely by vaccine, which ranged between 0 to 45 doses per day. The distribution and dispersion of doses administered for different vaccines for two districts are shown in Fig. 1a and b).

The doses administered per session for most of the vaccine doses were higher in Kangra district than Pune. The dosage administered per session for LPV, RVV, OPV and IPV were similar in both districts. There were fewer doses of BCG and HBV administered at these sessions, as most of these birth doses were given at the hospitals immediately after delivery. About 75–80% of the vaccination sessions had beneficiary turnout number less than the doses in one vaccine vial. In Kangra the overall turnout rate for vaccination sessions was 86% with BCG (81%), DPT (83%), HBV (80%), IPV (88%), measles (87%), measles-rubella (MR) (86%), OPV (86%), PCV (86%), LPV (87%), RVV (85%), and TT (85%). In Pune the overall turnout rate for vaccination sessions was 67% with BCG (64%), DPT (53%), HBV (70%), IPV (98%), Measles (68%), OPV (64%), LPV (72%), RVV (75%), and TT (64%). While 3–4 vaccination sessions monthly were conducted under each sub-centre in Pune, usually 1–2 sessions monthly in Kangra district.

*Vaccine wastage at the outreach session level:* The doses issued to the session sites and administered and vaccine usage and wastage rates for each vaccine not following open vial policy were calculated for the two districts (Table 1).

**Table 1:** Vaccine wastage by antigen at outreach session level in the two districts

Antigen	Vial size (doses/vial)	Doses administered (A)	Doses issued (B)	Vaccine usage rate C = (A/B) X 100	Vaccine wastage rate D = (100-C)	Vaccine wastage range^a^ (D, %)	Wastage Factor E = 100/ (100-D)
***Kangra***							
BCG	10	1220	1810	67.4	32.6	0–91	1.5
Measles	5	1592	2037	78.2	21.8	10–93.3	1.3
MR	10	1312	1378	95.2	4.8	0–92	1.1
RVV-5	5	3344	4336	77.1	22.9	15–85.7	1.3
RVV-10	10	2699	4259	63.4	36.6	10–90.8	1.6
RVV pooled	5/10	6043	8595	70.3	29.7	10–90.8	1.4
***Pune***							
BCG	10	1584	2110	75.1	24.9	25–93.3	1.3
Measles	5	3697	5091	72.6	27.4	25–95.7	1.4
RVV-5	5	1296	1731	74.9	25.1	8–64.6	1.3
RVV-10	10	3445	4200	82.0	18.0	20–72.8	1.2
RVV pooled	5/10	4741	5931	79.9	20.1	8–72.8	1.3

*RVV-5* Rotavirus vaccine, 2.5 ml, 5 doses per vial and *RVV-10* Rotavirus vaccine, 5 ml, 10 doses per vial

^a^The vaccine wastage levels at the outreach sessions across the different months

The overall wastage rates for BCG and pooled RVV was higher in Kangra district than Pune. The vaccine wastage rates for the outreach sessions for various vaccines (RVV, Measles, MR and BCG) and pooled for the districts are summarised in Table 2. It was observed that while 45% of the outreach sessions in Kangra had < 25% wastage rates for vaccines, 80% of the Pune sessions had similar wastage level.

**Table 2:** Wastage levels for various vaccines at the outreach sessions

District	WR level	WR for vaccines (%)				
		Pooled	RVV	Measles	MR	BCG
Kangra	< 25%	45	28.8	11.4	2.3	2.5
	26–50%	19.8	4.1	12.5	2	1.2
	> 50%	35.2	13.7	12.2	4.5	4.8
Pune	< 25%	80.2	33.5	37.5	–	9.2
	26–50%	4.6	1.8	1.8	–	1
	> 50%	15.2	5.3	2.1	–	7.8

*WR* Wastage rate, *RVV* Rotavirus vaccine, *MR* Measles and rubella vaccine, *Pooled* Pooled wastage rate for the district

### Vaccine wastage at the stores (CCPs) level

The vaccine usage and wastage rates for each vaccine for the CCPs were calculated using the doses issued for the sessions and administered on monthly basis. The summarised wastage rates for the vaccines and their ranges across various CCPs (pooled for the district including district, CHC and PHC stores) are reflected in Table 3. The wastage rates varied widely for different vaccines and between the districts. The vaccine wastage rates were > 25% for all vaccines in Kangra district. The wastage rates were 26–50% for most of the vaccines except OPV and fIPV which had wastage rates > 50%. The wastage rates were < 25% for HBV, Measles, OPV, LPV, TT, IPV and RVV-pooled in Pune. The wastage rates were 26–50% for BCG, DPT, fIPV and RVV-10 dose vials. No wastage of unopened vials was documented at the CCPs. The ranges of wastage for different vaccines at CCPs varied widely. The wastage for vaccines at facility and outreach levels are compared in Supplementary file 4.

**Table 3:** Vaccine wastage by antigen at cold chain store unit level in the two districts

Antigen	Vial size (doses/vial)	Doses administered (A)	Doses issued (B)	Vaccine usage rate C = (A/B) X 100 (%)	Vaccine wastage rate D = (100-C) (%)	Vaccine wastage range^a^ (D, %)	Wastage Factor E = 100/ (100-D)
***Kangra***							
BCG	10	33,741	53,614	62.9	37.1	5–83	1.6
DPT	10	87,325	128,532	67.9	32.1	5–80	1.5
HBV	10	3450	4952	69.7	30.3	5.5–70.6	1.4
Measles	5	45,747	67,459	67.8	32.2	0–81	1.5
MR	10	78,708	132,270	59.5	40.5	0–90	1.7
OPV	20	203,011	412,723	49.2	50.8	0–75	2.0
PCV	10	19,651	26,908	73.0	27.0	0–83	1.4
LPV	10	98,578	120,741	81.6	18.4	0–83	1.2
TT	10	32,599	49,480	65.9	34.1	4.8–76	1.5
IPV	10	13,142	20,553	63.9	36.1	0–77	1.6
fIPV	50	27,238	60,296	45.2	54.8	10–82	2.2
IPV pooled	10/50	40,380	80,849	49.9	50.1	0–82	2.0
RVV-5	5	40,278	56,758	71.0	29.0	0–75	1.4
RVV-10	10	73,107	109,516	66.8	33.2	0–82	1.5
RVV pooled	5/10	113,385	166,274	68.2	31.8	0–82	1.5
***Pune***							
BCG	10	12,121	18,664	64.9	35.1	0–87.5	1.5
DPT	10	13,884	18,603	74.6	25.4	0–78	1.3
HBV	10	7985	9955	80.2	19.8	6.6–80	1.2
Measels	5	22,313	28,506	78.3	21.7	0–75	1.3
OPV	20	80,787	94,308	85.7	14.3	0–74.3	1.2
LPV	10	36,432	41,985	86.8	13.2	0–78.6	1.2
TT	10	36,481	47,410	76.9	23.1	0–87.6	1.3
IPV	10	718	880	81.6	18.4	0–60	1.2
fIPV	50	5123	7010	73.1	26.9	9–76	1.4
IPV pooled	10/50	5841	7890	74.0	26.0	0–76	1.4
RVV-5	5	10,335	12,570	82.2	17.8	0–88	1.2
RVV-10	10	21,941	29,524	74.3	25.7	0–90	1.3
RVV pooled	5/10	32,276	42,094	76.7	23.3	0–90	1.3

*IPV* Inactivated polio vaccine standard 0.5 ml/dose intramuscular, 10 doses per vial, *fIPV* Fractional Inactivated polio vaccine, 0.1 ml/dose intradermal, 50 doses per vial, *RVV-5* Rotavirus vaccine, 2.5 ml, 5 doses per vial and *RVV-10* Rotavirus vaccine, 5 ml, 10 doses per vial

^a^The vaccine wastage levels at the cold chain points across the different months

### Vaccine wastage with transition of vial dosage sizes

At the cold chain point level, with transition from 5- to 10-doses of RVV vials, the wastage rate increased both in Kangra (29 to 33.2%) and Pune (17.8 to 25.7%) (Table 3). The wastage rate for RVV at outreach session level increased with transition from 5- to 10-doses vial in Kangra (22.9 to 36.6%), while it declined in Pune (25.1 to 18%) (Table 1).

### Vaccine wastage with transition from IPV to fIPV practice

With transition of IPV dosage from 0.5 ml to fractional 0.1 ml (fIPV), the wastage rates at CCP level increased sizably in Kangra (36.1 to 54.8%) and marginally in Pune (18.4 to 26.9%) (Table 3).

### Vaccine wastage according to the vaccine types and vial sizes

The wastage rates for vaccines according to the type (liquid or lyophilised), reuse type (open vial policy), and vial sizes (number of doses) for oral and injectable routes for two districts for cold chain stores and outreach levels are summarised in Table 4.

**Table 4:** Wastage rates for antigens according to the composition and vial size

Type/Form	Kangra district		Pune district	
	Cold chain point Site WR (%)	Outreach session sites WR (%)	Cold chain point Site WR (%)	Outreach session sites WR (%)
I: Type of Vaccine				
Liquid	42.2	29.7^a^	17.4	20.1^a^
Lyophilized	35.3	26.7^b^	25.3	26.1^b^
II: Reuse Type				
Open vial	42.2	–	17.4	–
Single use vial	35.3	22.2^c^	25.3	24.1^c^
III: Vial Size-Oral				
5 doses	29.1	22.9^d^	17.8	25.1^d^
10 doses	33.2	36.6^e^	25.7	18.0^e^
20 doses	50.8	–	14.3	–
Oral pooled	45.3	29.7	17.1	20.1
IV: Vial Size- Injectable				
5 doses	37.7	21.8^f^	21.7	27.4^f^
10 doses	28.7	18.7^g^	21.4	24.9^g^
50 doses (fIPV)	54.8	–	26.9	–
Injectable pooled	33.8	19.7	21.7	26.1

*WR* wastage rate in percentages

^a^The wastage rate for liquid vaccine at outreach sessions includes RVV only

^b^The wastage rate for lyophilized vaccines at outreach sessions includes BCG, Measles and MR vaccines

^c^The wastage rate for single use vial vaccines at outreach sessions includes BCG, Measles, MR and RVV (5/10 doses) vaccines

^d^The wastage rate for 5 doses vial vaccines at outreach sessions includes Measles and RVV (5 doses) vaccines

^e^The wastage rate for 10 doses vial vaccines at outreach sessions includes BCG, MR and RVV (10 doses) vaccines

^f^The wastage rate for 5 doses injectable vial vaccine at outreach sessions includes measles vaccine

^g^The wastage rate for 10 doses injectable vial vaccines at outreach sessions includes BCG and MR vaccines

Generally the wastage rates for majority of the vaccines in Kangra district were higher than Pune, except for five dose (both oral and injectable) and 10 dose (only injectable) vials. The higher wastage rates in Pune district for five dose vials were due to measles and RVV. The lower wastage rate in Kangra district for 10 dose injectable vials was due to lesser wastage of MR vaccine at outreach sessions (Table 1). While the pooled wastage rates for liquid vaccines were higher than the lyophilized vaccines in Kangra, the reverse pattern was observed in Pune (Supplementary files 5 and 6). The wastage rated for single use vials and pooled oral vaccines were comparable for the two districts. For the five dose vials, the wastage was lesser in Kangra than Pune.

### Perceptions and practices regarding vaccine wastage

Almost all health functionaries indicated that the vaccine wastage increased with transition of 5 to 10 doses vials of RVV and IM IPV to f-IPV in both districts. To minimise the wastage, the store-in-charges had been issuing the vials according to the anticipated beneficiary numbers. Majority of the vaccinators and CCHs from Kangra reported that the outreach sessions were rescheduled/reorganised to minimise the wastages. The MOs and DIO were aware about the session rescheduling/reorganisations made by the vaccinators. Few vaccinators from sites with high beneficiary load, didn’t find much difference in the wastage with the change in vial. The statements from various stakeholders are presented in the Supplementary document (Supplementary file 7).

## Discussion

This report attempted to document the vaccine wastage status for the vaccines in UIP in Kangra and Pune districts representing two different geographies, the hilly and plain regions. The use of two different sizes of RVV vials provided an opportunity for documenting the change in wastage rates. Additionally the transition from regular to fraction dose of IPV in Kangra during the observation period was also captured. We estimated the wastage for UIP vaccines at CCPs and outreach session sites during 2 year period. This is the first report from India on wastage rate of RVV with both 5 and 10 dosage vial package versions. Additionally this is the first report from India on wastage for PCV and transition of IPV to fIPV dosage under UIP.

The present study documented higher wastage rates for several vaccines than the recommendation by MoHFW. In Kangra, the wastage rates for OPV and fIPV were above 50%, for most of the other vaccines except LPV were in the range of 26–50%. For most of the vaccines the wastage was >10% of the prescribed range, except Measles, LPV and RVV-5. In Pune, the wastage rates for most of the vaccines were < 25% except for BCG, DPT, fIPV, and RVV-10. For majority of the vaccines the wastage was within 10% of the prescribed range, except DPT, TT, and fIPV. For Measles and RVV-5, the wastage was lower than the prescribed range. Higher overall vaccine wastage rates in Kangra compared to Pune may be explained by the target population base, density and beneficiary load for the sessions. The wastage rates for outreach sessions for the five dose vials were higher in Pune district than Kangra, which may be explained by the beneficiary number per sessions. No wastage of unopened vials indicated good practices for storage, logistics and stock management at the CCPs.

Rise in the wastage rate with transition from RVV 5 doses to 10 doses vial in both districts can be explained by the beneficiary case load. With rising institutional deliveries, fewer beneficiaries for BCG vaccine at outreach session level may explain little higher wastage. The wastage rates for vaccines under open vial policy like DPT, fIPV may be higher due to lower number of beneficiaries per session (outreach and facility based) within the usable window period of 4 weeks in the same session area. Despite higher wastage for fIPV (50 doses) dose practice, higher number of beneficiaries would have been vaccinated from the unit vial compared to IPV (10 doses) dose practice. Using the opened vaccine vial for sessions other than the session where it is opened may reduce the wastage of these multidose vaccine vials under open vial policy. Most of the stakeholders from both districts reported increased RVV wastage with change of vial size (5 to 10 doses vials). Recognising the increased wastage with 10 doses vial, the health functionaries had reorganised the sessions to increase the pooled beneficiaries number and reduce the wastage.

The available reports on vaccine wastage from outreach sessions, CCPs and immunization clinics of tertiary care facilities are summarised in Table 5. The wastage rates for Himachal Pradesh were higher compared to several other states. Compared to the earlier report, the wastage rates for most of the vaccines have declined during the current study period [2]. Similarly the wastage rates for several vaccines in Pune are lower than the earlier report from Maharashtra [2]. The wastage rates at outreach and cold chain store (including outreach sessions and facility sessions) were comparable as per the available reports.

**Table 5:** Vaccine wastage rates reported from India and globally

Author	State	Level	Wastage rates for vaccines (%)										
			BCG	OPV	DPT	HBV	LPV	DT	TT	IPV	Measles	MR	MMR
**India**													
Unicef (2010) [2]	UP	OR	58	40	19	–	–		20		26	–	
	HP	OR	65	75	58	57	–		53		58	–	
	TN	OR	65	46	20	30	–		32		35	–	
	MH	OR	54	51	35	37	–		55		44	–	
	AS	OR	68	56	34	–	–		41		41	–	
	Pooled	OR	60.9	47.4	26.8	33.1	–		33.7		35.1	–	
Chinnakali P et al. (2012) [6]	Delhi	OR	70.9	48.1	38.6	–		57.3	62.8		39.9	–	37.5
Mukherjee et al. (2013) [7]	UP	Store^a^	50.8	35.7	19.6			46.0	54.1		31.7		
	Bihar	Store^a^	58.8	46.9	24.1			33.6	34.1		22.3		
	WB	Store^a^	45.5	66.7	60.5			46.2	55.4		28.3		
	Goa	Store^a^	56.3	36.3	20.8			37.2	29.3		28.4		
	Pooled	Store^a^	49.3	52.7	38.9			39.1	48.0		27.9		
Mehta S et al. (2013) [8]	GJ	Store^a^	45	25	16	21					25		
Shah HD et al. (2016) [9]	GJ	Store^a^	42.5		40.9		30.7		35.8		40.4		
Patel PB et al. (2016) [10]	GJ	Store^a^		13.6	8.5								
Duttagupta C et al. (2017) [11]	KA	Store^a^	64.6	41.4	50.2		24.5				32.5		
Gupta V et al. (2015) [12]	HR	Store^b^	77.9	28.9	46.7	38.6	7.4		36.8		41.2		
Daya P et al. (2015) [13]	PD	Store^b^		2.4	8.4	5.3			4.2		46.5		1.4
Sharma G et al. (2016) [14]	RJ	Store^b^	21.3	29.4	12.7	9.2	9.3				27.6		
Parmar D et al. (2016) [4]	MH	Store^b^	22.9	6.7	3.4	6.5			6.13		11		
Tiwari R et al. (2017) [15]	MP	Store^b^	20.7	14.6	15.6	10.5	5.2	–	7.0	10.4	21.6		
**Other countries**													
Guichard S et al. (2010) [16]	BG	Store^a^	84.4		45.1				34.8		68.6		
Wallace AS et al. (2017) [17]	Nigeria	Store^a^	22.5	22.5	18	21			28		27		
		OR	30	35	21	22			30		23		
Usuf E et al. (2018) [18]	Gambia	Store^a^	54.9	4.4	0.1	1.9	2.5		2.0	5.1	15.6		

*AS* Assam, *BG* Bangladesh, *GJ* Gujarat, *HP* Himachal Pradesh, *HR* Haryana, *KA* Karnataka, *MH* Maharashtra, *MP* Madhya Pradesh, *PD* Puducherry, *RJ* Rajasthan, *TN* Tamil Nadu, *UP* Uttar Pradesh, *WB* West Bengal

^a^Vaccine store and outreach combined

^b^Single facility immunization clinic

The reported vaccine wastage rates from other developing countries varied widely. The vaccine wastage rates from Bangladesh (34.8 to 84.4%) are comparable to Indian figures [16].. The wastage rates in Nigeria were in the range of 21–35% at outreach sessions and 18–27% at the stores [17]. The wastage rates were lower in Gambia for most of the vaccines, except the BCG (54.9%) [18]. In Gambia the wastage of RVV (single dose vials) has been reported to be 5.2%, mostly due to expiry [18].

There have been several efforts to make immunization program cost effective through appropriate adjustment in vaccine logistics, operation including session planning, vial reuse policy, vial size package, and vaccinator practice [19]. It was estimated that with transition from 10- to 5-dose vials of IPV the open vial wastage would be reduced by nearly half in all countries; Bangladesh by 56% (from 0.25 to 0.11), India by 53% (from 0.17 to 0.08), Mozambique by 53% (from 0.13 to 0.06), and Uganda by 44% (from 0.09 to 0.04) [20].

The two states (Himachal Pradesh and Maharashtra) are common with the Unicef vaccine wastage study in five states [2]. The vaccine wastage rates for most of the vaccines in both states have decreased overtime, which may be due to better organisation and logistics management, improved supervision and monitoring and open vial policy adoption. There has been wide variation in the vaccine wastage across the states and most of them have been from urban or tertiary care facilities. Limited information on vaccine wastage at outreach levels is available from different states in India to better understand the determinants. In our study, with transition from smaller to larger dose vial size, the wastage rate increases as documented for RVV and IPV in the current study. Reducing vial size has been an appealing strategy to reduce wastage, but cost-benefit analysis should be conducted considering the logistics and cold chain footprint, especially the costlier vaccines.

This study had several limitations. The study included only two districts and selected facilities in the districts. Some of the CCPs and outreach sessions had incomplete records, which may have introduced bias in the estimates. For the vaccines under open vial policy (DPT, LPV, HBV, TT, IPV, OPV and PCV), the doses returned from the session sites were not recorded. We used the doses issues and doses administered for estimation of the wastage, which may have bias in estimates. The period of record available for MR and PCV were relatively small and for one district only.

## Conclusions

The study conducted in two different geography and population contexts in India documented that the vaccine wastage rates in both districts were relatively higher than the national program assumptions. The hilly district (Kangra) had relatively higher wastage for all vaccines compared to the plain district (Pune). The change in vial size for RVV from 5- to 10 doses increased the wastage in both districts. The transition from intramuscular IPV to intradermal fIPV has also increased the wastage rate, but the children vaccinated from one vial had also increased. The UIP in India face pressure to minimize the wastage and improve coverage with the available vaccine. With introduction of newer vaccines the coverage and vaccine wastage needs to be monitored for informing the program and operation. Close monitoring the wastage rates at different levels including the service delivery points; facilities with different case load and outreach levels in different geographies with various microplan types and stores at different levels. India provides opportunity for documenting the vaccine wastage across different contexts and lessons for appropriate programmatic action.

## Supplementary information

**Additional file 1: Supplementary file 1.** Vaccine wastage documentation tool at sub-centres and session sites

**Additional file 2: Supplementary file 2.** Vaccine wastage documentation tool at facility level

**Additional file 3: Supplementary file 3.** In-depth interview guides for stakeholders. **Supplementary file 3.1**: In-depth interview guide- Cold chain handler. **Supplementary file 3.2**: In-depth interview guide- Vaccinator . **Supplementary file 3.3**: In-depth interview guide- Store in-charge. **Supplementary file 3.4**: In-depth interview guide-Medical Officer. **Supplementary file 3.5**: In-depth interview guide-District Immunization Officer

**Additional file 4: Table S1.** Wastage rates (as percentage) according to the type of sessions

**Additional file 5: Table S2.** The trend of vaccine wastage across months in Kangra district (pooled for the district)

**Additional file 6: Table S3.** The trend of vaccine wastage across months in Pune district (pooled for the district)

**Additional file 7: Supplementary file 7.** Statements of stakeholders regarding the vaccine wastage and strategies to minimise wastage

## Data Availability

The datasets collected and analysed for the current study are available from the corresponding author on request.

## Abbreviations

BCG: Bacillus Calmette–Guérin
CHC: Community health centres
DH: District hospital
DPT: Diptheria-pertusis-tetanus
fIPV: Fractional Inactivated polio vaccine
GAVI: The Global Alliance for Vaccines and Immunization
HBV: Hepatitis B vaccine
IPV: Inactivated polio vaccine
LPV: Liquid pentavalent vaccine
MOHFW: Ministry of Health and Family Welfare
MR: Measles-rubella vaccine
MySQL: An open-source relational database management system
OPV: Oral polio vaccine
PCV: Pneumococcal conjugate vaccine
PHC: Primary health centres
PHP: Personal Home Page (a server language for web development)
RH: Rural hospital
SDH: Sub-district hospital
TT: Tetanus toxoid
RVV: Rotavirus vaccine
UIP: Universal Immunization program
WHO: World Health organisation
