# Supplementary material for: Documentation of vaccine wastage in two different geographic contexts under the universal immunization program in India
Source: BMC Public Health. 2020 Apr 25;20:556. doi: 10.1186/s12889-020-08637-1 (PMC7183620; doi:10.1186/s12889-020-08637-1)
Supplement: Supplementary file 1 — Additional file 1: Supplementary file 1. Vaccine wastage documentation tool at sub-centres and session sites [file 12889_2020_8637_MOESM1_ESM.docx]

| **UNIQUE ID** |  |
| --- | --- |

**Documentation of vaccine wastage under the universal immunization program in the districts of Kangra (Himachal Pradesh) and Pune (Maharashtra)**

**Vaccine Utilization Documentation Tool (VUDT)**

**(For Sub-Centers and Immunization Session Sites)**

| - 1. **District Name** | | | **Kangra Pune** |
| --- | --- | --- | --- |
| - 1. **Block Name** | | |  |
| - 1. **Name of the Facility/Session Site** | | |  |
| - 1. **Name of the PHC** | | |  |
| **1.5 Type/Level of Vaccine Store**  1- District, 2- SDH/RH/ZH; 3-CHC;  4- PHC/UPHC; 5-Sub center  6- Outreach session 9- Other | | | If other, specify ………………………………… |
| **1.6 Date of assessment/visit** | | |  |
| **1.7** | **Persons/ staff met during the visit** (write only designations, not names) | 1. |  |
|  |  | 2. |  |
|  |  | 3. |  |
| **1.8** | **Documents reviewed**  (source documents for data collection)  (mark ‘X’ for the reviewed documents) | 1. Vaccinator’s Logistics Diary |  |
|  |  | 2. Vaccination register |  |
|  |  | 3. Due list/ Tally sheets |  |
|  |  | 4. Microplan |  |
|  |  | 5. Monthly report register |  |
|  |  | 6. Other …………………………. |  |

**Section A: Population and session information**

**Q2. Please record the area and target population served**

| **Q no** | **Question** | | **Total (n)** | **Year of estimation** |
| --- | --- | --- | --- | --- |
| **2.1** | **Population served by the sub-center** | |  |  |
| 2.1.1 | Total population served | |  |  |
| 2.1.2 | Annual birth cohort | |  |  |
| 2.1.3 | Total pregnant women (registered annually) | |  |  |
| 2.2 | **Population served by the vaccination session sites** | | | |
|  | **Question** | **Session site/area** | **Population (total)** | **Schedule (day & week)** |
| 2.2.1 | Sub-center session |  |  |  |
| 2.2.2 | Outreach session 1 |  |  |  |
| 2.2.3 | Outreach session 2 |  |  |  |
| 2.2.4 | Outreach session 3 |  |  |  |
| 2.2.5 | Outreach session 4 |  |  |  |
| 2.2.6 | Outreach session 5 |  |  |  |
| 2.2.7 | Outreach session 6 |  |  |  |
| 2.2.8 | Outreach session 7 |  |  |  |

**Section B: Vaccine receipt and return from the session sites**

**Q 3. Please record the vaccines received and returned from the sessions for each month during Jan 2016- Dec 2017.**

**3.1 Please record the vaccines received and returned from the session sites under the sub-center (Month January 2016)**

| **Q no** | **Vaccine name** | **Session 1** | | | | **Session 2** | | | | **Session 3** | | | | | **Session 4** | | | | |
| --- | --- | --- | --- | --- | --- | --- | --- | --- | --- | --- | --- | --- | --- | --- | --- | --- | --- | --- | --- |
|  |  | **Received vaccines** | | **Returned vaccine** | | **Received vaccines** | | **Returned vaccine** | | **Received vaccines** | | **Returned vaccine** | | | **Received vaccines** | | **Returned vaccine** | | |
|  |  | Unopened (doses) | Opened (doses) | Unopened (doses) | Opened (doses) | Unopened (doses) | Opened (doses) | Unopened (doses) | Opened (doses) | Unopened (doses) | Opened (doses) | Unopened (doses) | Opened (doses) | Unopened (doses) | | Opened (doses) | Unopened (doses) | Opened (doses) |  |
| **3.1.1** | OPV |  |  |  |  |  |  |  |  |  |  |  |  |  | |  |  |  |  |
| **3.1.2** | HBV |  |  |  |  |  |  |  |  |  |  |  |  |  | |  |  |  |  |
| **3.1.3** | BCG |  |  |  |  |  |  |  |  |  |  |  |  |  | |  |  |  |  |
| **3.1.4** | Penta |  |  |  |  |  |  |  |  |  |  |  |  |  | |  |  |  |  |
| **3.1.5** | DPT |  |  |  |  |  |  |  |  |  |  |  |  |  | |  |  |  |  |
| **3.1.6** | Rota |  |  |  |  |  |  |  |  |  |  |  |  |  | |  |  |  |  |
| **3.1.7** | IPV |  |  |  |  |  |  |  |  |  |  |  |  |  | |  |  |  |  |
| **3.1.8** | Measles |  |  |  |  |  |  |  |  |  |  |  |  |  | |  |  |  |  |
| **3.1.9** | MR |  |  |  |  |  |  |  |  |  |  |  |  |  | |  |  |  |  |
| **3.1.10** | TT |  |  |  |  |  |  |  |  |  |  |  |  |  | |  |  |  |  |
| **3.1.11** | PCV |  |  |  |  |  |  |  |  |  |  |  |  |  | |  |  |  |  |
| **3.1.12** | JE |  |  |  |  |  |  |  |  |  |  |  |  |  | |  |  |  |  |
| **3.1.13** | MMR |  |  |  |  |  |  |  |  |  |  |  |  |  | |  |  |  |  |

**3.2 Please record the vaccines received and returned from the session sites under the sub-center (Month February 2016)**

| **Q no** | **Vaccine name** | **Session 1** | | | | **Session 2** | | | | **Session 3** | | | | **Session 4** | | | | |
| --- | --- | --- | --- | --- | --- | --- | --- | --- | --- | --- | --- | --- | --- | --- | --- | --- | --- | --- |
|  |  | **Received vaccines** | | **Returned vaccine** | | **Received vaccines** | | **Returned vaccine** | | **Received vaccines** | | **Returned vaccine** | | **Received vaccines** | | **Returned vaccine** | | |
|  |  | Unopened (doses) | Opened (doses) | Unopened (doses) | Opened (doses) | Unopened (doses) | Opened (doses) | Unopened (doses) | Opened (doses) | Unopened (doses) | Opened (doses) | Unopened (doses) | Opened (doses) | Unopened (doses) | Opened (doses) | Unopened (doses) | Opened (doses) |  |
| **3.2.1** | OPV |  |  |  |  |  |  |  |  |  |  |  |  |  |  |  |  |  |
| **3.2.2** | HBV |  |  |  |  |  |  |  |  |  |  |  |  |  |  |  |  |  |
| **3.2.3** | BCG |  |  |  |  |  |  |  |  |  |  |  |  |  |  |  |  |  |
| **3.2.4** | Penta |  |  |  |  |  |  |  |  |  |  |  |  |  |  |  |  |  |
| **3.2.5** | DPT |  |  |  |  |  |  |  |  |  |  |  |  |  |  |  |  |  |
| **3.2.6** | Rota |  |  |  |  |  |  |  |  |  |  |  |  |  |  |  |  |  |
| **3.2.7** | IPV |  |  |  |  |  |  |  |  |  |  |  |  |  |  |  |  |  |
| **3.2.8** | Measles |  |  |  |  |  |  |  |  |  |  |  |  |  |  |  |  |  |
| **3.2.9** | MR |  |  |  |  |  |  |  |  |  |  |  |  |  |  |  |  |  |
| **3.2.10** | TT |  |  |  |  |  |  |  |  |  |  |  |  |  |  |  |  |  |
| **3.2.11** | PCV |  |  |  |  |  |  |  |  |  |  |  |  |  |  |  |  |  |
| **3.2.12** | JE |  |  |  |  |  |  |  |  |  |  |  |  |  |  |  |  |  |
| **3.2.13** | MMR |  |  |  |  |  |  |  |  |  |  |  |  |  |  |  |  |  |

**3.3 Please record the vaccines received and returned from the session sites under the sub-center (Month March 2016)**

| **Q no** | **Vaccine name** | **Session 1** | | | | **Session 2** | | | | **Session 3** | | | | **Session 4** | | | | |
| --- | --- | --- | --- | --- | --- | --- | --- | --- | --- | --- | --- | --- | --- | --- | --- | --- | --- | --- |
|  |  | **Received vaccines** | | **Returned vaccine** | | **Received vaccines** | | **Returned vaccine** | | **Received vaccines** | | **Returned vaccine** | | **Received vaccines** | | **Returned vaccine** | | |
|  |  | Unopened (doses) | Opened (doses) | Unopened (doses) | Opened (doses) | Unopened (doses) | Opened (doses) | Unopened (doses) | Opened (doses) | Unopened (doses) | Opened (doses) | Unopened (doses) | Opened (doses) | Unopened (doses) | Opened (doses) | Unopened (doses) | Opened (doses) |  |
| **3.3.1** | OPV |  |  |  |  |  |  |  |  |  |  |  |  |  |  |  |  |  |
| **3.3.2** | HBV |  |  |  |  |  |  |  |  |  |  |  |  |  |  |  |  |  |
| **3.3.3** | BCG |  |  |  |  |  |  |  |  |  |  |  |  |  |  |  |  |  |
| **3.3.4** | Penta |  |  |  |  |  |  |  |  |  |  |  |  |  |  |  |  |  |
| **3.3.5** | DPT |  |  |  |  |  |  |  |  |  |  |  |  |  |  |  |  |  |
| **3.3.6** | Rota |  |  |  |  |  |  |  |  |  |  |  |  |  |  |  |  |  |
| **3.3.7** | IPV |  |  |  |  |  |  |  |  |  |  |  |  |  |  |  |  |  |
| **3.3.8** | Measles |  |  |  |  |  |  |  |  |  |  |  |  |  |  |  |  |  |
| **3.3.9** | MR |  |  |  |  |  |  |  |  |  |  |  |  |  |  |  |  |  |
| **3.3.10** | TT |  |  |  |  |  |  |  |  |  |  |  |  |  |  |  |  |  |
| **3.3.11** | PCV |  |  |  |  |  |  |  |  |  |  |  |  |  |  |  |  |  |
| **3.3.12** | JE |  |  |  |  |  |  |  |  |  |  |  |  |  |  |  |  |  |
| **3.3.13** | MMR |  |  |  |  |  |  |  |  |  |  |  |  |  |  |  |  |  |

**3.4 Please record the vaccines received and returned from the session sites under the sub-center (Month April 2016)**

| **Q no** | **Vaccine name** | **Session 1** | | | | **Session 2** | | | | **Session 3** | | | | **Session 4** | | | | |
| --- | --- | --- | --- | --- | --- | --- | --- | --- | --- | --- | --- | --- | --- | --- | --- | --- | --- | --- |
|  |  | **Received vaccines** | | **Returned vaccine** | | **Received vaccines** | | **Returned vaccine** | | **Received vaccines** | | **Returned vaccine** | | **Received vaccines** | | **Returned vaccine** | | |
|  |  | Unopened (doses) | Opened (doses) | Unopened (doses) | Opened (doses) | Unopened (doses) | Opened (doses) | Unopened (doses) | Opened (doses) | Unopened (doses) | Opened (doses) | Unopened (doses) | Opened (doses) | Unopened (doses) | Opened (doses) | Unopened (doses) | Opened (doses) |  |
| **3.4.1** | OPV |  |  |  |  |  |  |  |  |  |  |  |  |  |  |  |  |  |
| **3.4.2** | HBV |  |  |  |  |  |  |  |  |  |  |  |  |  |  |  |  |  |
| **3.4.3** | BCG |  |  |  |  |  |  |  |  |  |  |  |  |  |  |  |  |  |
| **3.4.4** | Penta |  |  |  |  |  |  |  |  |  |  |  |  |  |  |  |  |  |
| **3.4.5** | DPT |  |  |  |  |  |  |  |  |  |  |  |  |  |  |  |  |  |
| **3.4.6** | Rota |  |  |  |  |  |  |  |  |  |  |  |  |  |  |  |  |  |
| **3.4.7** | IPV |  |  |  |  |  |  |  |  |  |  |  |  |  |  |  |  |  |
| **3.4.8** | Measles |  |  |  |  |  |  |  |  |  |  |  |  |  |  |  |  |  |
| **3.4.9** | MR |  |  |  |  |  |  |  |  |  |  |  |  |  |  |  |  |  |
| **3.4.10** | TT |  |  |  |  |  |  |  |  |  |  |  |  |  |  |  |  |  |
| **3.4.11** | PCV |  |  |  |  |  |  |  |  |  |  |  |  |  |  |  |  |  |
| **3.4.12** | JE |  |  |  |  |  |  |  |  |  |  |  |  |  |  |  |  |  |
| **3.4.13** | MMR |  |  |  |  |  |  |  |  |  |  |  |  |  |  |  |  |  |

**3.5 Please record the vaccines received and returned from the session sites under the sub-center (Month May 2016)**

| **Q no** | **Vaccine name** | **Session 1** | | | | **Session 2** | | | | **Session 3** | | | | **Session 4** | | | | |
| --- | --- | --- | --- | --- | --- | --- | --- | --- | --- | --- | --- | --- | --- | --- | --- | --- | --- | --- |
|  |  | **Received vaccines** | | **Returned vaccine** | | **Received vaccines** | | **Returned vaccine** | | **Received vaccines** | | **Returned vaccine** | | **Received vaccines** | | **Returned vaccine** | | |
|  |  | Unopened (doses) | Opened (doses) | Unopened (doses) | Opened (doses) | Unopened (doses) | Opened (doses) | Unopened (doses) | Opened (doses) | Unopened (doses) | Opened (doses) | Unopened (doses) | Opened (doses) | Unopened (doses) | Opened (doses) | Unopened (doses) | Opened (doses) |  |
| **3.5.1** | OPV |  |  |  |  |  |  |  |  |  |  |  |  |  |  |  |  |  |
| **3.5.2** | HBV |  |  |  |  |  |  |  |  |  |  |  |  |  |  |  |  |  |
| **3.5.3** | BCG |  |  |  |  |  |  |  |  |  |  |  |  |  |  |  |  |  |
| **3.5.4** | Penta |  |  |  |  |  |  |  |  |  |  |  |  |  |  |  |  |  |
| **3.5.5** | DPT |  |  |  |  |  |  |  |  |  |  |  |  |  |  |  |  |  |
| **3.5.6** | Rota |  |  |  |  |  |  |  |  |  |  |  |  |  |  |  |  |  |
| **3.5.7** | IPV |  |  |  |  |  |  |  |  |  |  |  |  |  |  |  |  |  |
| **3.5.8** | Measles |  |  |  |  |  |  |  |  |  |  |  |  |  |  |  |  |  |
| **3.5.9** | MR |  |  |  |  |  |  |  |  |  |  |  |  |  |  |  |  |  |
| **3.5.10** | TT |  |  |  |  |  |  |  |  |  |  |  |  |  |  |  |  |  |
| **3.5.11** | PCV |  |  |  |  |  |  |  |  |  |  |  |  |  |  |  |  |  |
| **3.5.12** | JE |  |  |  |  |  |  |  |  |  |  |  |  |  |  |  |  |  |
| **3.5.13** | MMR |  |  |  |  |  |  |  |  |  |  |  |  |  |  |  |  |  |

**3.6 Please record the vaccines received and returned from the session sites under the sub-center (Month June 2016)**

| **Q no** | **Vaccine name** | **Session 1** | | | | **Session 2** | | | | **Session 3** | | | | **Session 4** | | | |
| --- | --- | --- | --- | --- | --- | --- | --- | --- | --- | --- | --- | --- | --- | --- | --- | --- | --- |
|  |  | **Received vaccines** | | **Returned vaccine** | | **Received vaccines** | | **Returned vaccine** | | **Received vaccines** | | **Returned vaccine** | | **Received vaccines** | | **Returned vaccine** | |
|  |  | Unopened (doses) | Opened (doses) | Unopened (doses) | Opened (doses) | Unopened (doses) | Opened (doses) | Unopened (doses) | Opened (doses) | Unopened (doses) | Opened (doses) | Unopened (doses) | Opened (doses) | Unopened (doses) | Opened (doses) | Unopened (doses) | Opened (doses) |
| **3.6.1** | OPV |  |  |  |  |  |  |  |  |  |  |  |  |  |  |  |  |
| **3.6.2** | HBV |  |  |  |  |  |  |  |  |  |  |  |  |  |  |  |  |
| **3.6.3** | BCG |  |  |  |  |  |  |  |  |  |  |  |  |  |  |  |  |
| **3.6.4** | Penta |  |  |  |  |  |  |  |  |  |  |  |  |  |  |  |  |
| **3.6.5** | DPT |  |  |  |  |  |  |  |  |  |  |  |  |  |  |  |  |
| **3.6.6** | Rota |  |  |  |  |  |  |  |  |  |  |  |  |  |  |  |  |
| **3.6.7** | IPV |  |  |  |  |  |  |  |  |  |  |  |  |  |  |  |  |
| **3.6.8** | Measles |  |  |  |  |  |  |  |  |  |  |  |  |  |  |  |  |
| **3.6.9** | MR |  |  |  |  |  |  |  |  |  |  |  |  |  |  |  |  |
| **3.6.10** | TT |  |  |  |  |  |  |  |  |  |  |  |  |  |  |  |  |
| **3.6.11** | PCV |  |  |  |  |  |  |  |  |  |  |  |  |  |  |  |  |
| **3.6.12** | JE |  |  |  |  |  |  |  |  |  |  |  |  |  |  |  |  |
| **3.6.13** | MMR |  |  |  |  |  |  |  |  |  |  |  |  |  |  |  |  |

**3.7 Please record the vaccines received and returned from the session sites under the sub-center (Month July 2016)**

| **Q no** | **Vaccine name** | **Session 1** | | | | **Session 2** | | | | **Session 3** | | | | **Session 4** | | | | |
| --- | --- | --- | --- | --- | --- | --- | --- | --- | --- | --- | --- | --- | --- | --- | --- | --- | --- | --- |
|  |  | **Received vaccines(doses)** | | **Returned vaccine (doses)** | | **Received vaccines(doses)** | | **Returned vaccine (doses)** | | **Received vaccines(doses)** | | **Returned vaccine (doses)** | | **Received vaccines(doses)** | | **Returned vaccine (doses)** | | |
|  |  | Unopen | Open | Unopen | Open | Unopen | Open | Unopen | Open | Unopen | Open | Unopen | Open | Unopen | Open | Unopen | Open |  |
| **3.7.1** | OPV |  |  |  |  |  |  |  |  |  |  |  |  |  |  |  |  |  |
| **3.7.2** | HBV |  |  |  |  |  |  |  |  |  |  |  |  |  |  |  |  |  |
| **3.7.3** | BCG |  |  |  |  |  |  |  |  |  |  |  |  |  |  |  |  |  |
| **3.7.4** | Penta |  |  |  |  |  |  |  |  |  |  |  |  |  |  |  |  |  |
| **3.7.5** | DPT |  |  |  |  |  |  |  |  |  |  |  |  |  |  |  |  |  |
| **3.7.6** | Rota |  |  |  |  |  |  |  |  |  |  |  |  |  |  |  |  |  |
| **3.7.7** | IPV |  |  |  |  |  |  |  |  |  |  |  |  |  |  |  |  |  |
| **3.7.8** | Measles |  |  |  |  |  |  |  |  |  |  |  |  |  |  |  |  |  |
| **3.7.9** | MR |  |  |  |  |  |  |  |  |  |  |  |  |  |  |  |  |  |
| **3.7.10** | TT |  |  |  |  |  |  |  |  |  |  |  |  |  |  |  |  |  |
| **3.7.11** | PCV |  |  |  |  |  |  |  |  |  |  |  |  |  |  |  |  |  |
| **3.7.12** | JE |  |  |  |  |  |  |  |  |  |  |  |  |  |  |  |  |  |
| **3.7.13** | MMR |  |  |  |  |  |  |  |  |  |  |  |  |  |  |  |  |  |

**3.8 Please record the vaccines received and returned from the session sites under the sub-center (Month August 2016)**

| **Q no** | **Vaccine name** | **Session 1** | | | | **Session 2** | | | | **Session 3** | | | | **Session 4** | | | | |
| --- | --- | --- | --- | --- | --- | --- | --- | --- | --- | --- | --- | --- | --- | --- | --- | --- | --- | --- |
|  |  | **Received vaccines(doses)** | | **Returned vaccine (doses)** | | **Received vaccines(doses)** | | **Returned vaccine (doses)** | | **Received vaccines(doses)** | | **Returned vaccine (doses)** | | **Received vaccines(doses)** | | **Returned vaccine (doses)** | | |
|  |  | Unopen | Open | Unopen | Open | Unopen | Open | Unopen | Open | Unopen | Open | Unopen | Open | Unopen | Open | Unopen | Open |  |
| **3.8.1** | OPV |  |  |  |  |  |  |  |  |  |  |  |  |  |  |  |  |  |
| **3.8.2** | HBV |  |  |  |  |  |  |  |  |  |  |  |  |  |  |  |  |  |
| **3.8.3** | BCG |  |  |  |  |  |  |  |  |  |  |  |  |  |  |  |  |  |
| **3.8.4** | Penta |  |  |  |  |  |  |  |  |  |  |  |  |  |  |  |  |  |
| **3.8.5** | DPT |  |  |  |  |  |  |  |  |  |  |  |  |  |  |  |  |  |
| **3.8.6** | Rota |  |  |  |  |  |  |  |  |  |  |  |  |  |  |  |  |  |
| **3.8.7** | IPV |  |  |  |  |  |  |  |  |  |  |  |  |  |  |  |  |  |
| **3.8.8** | Measles |  |  |  |  |  |  |  |  |  |  |  |  |  |  |  |  |  |
| **3.8.9** | MR |  |  |  |  |  |  |  |  |  |  |  |  |  |  |  |  |  |
| **3.8.10** | TT |  |  |  |  |  |  |  |  |  |  |  |  |  |  |  |  |  |
| **3.8.11** | PCV |  |  |  |  |  |  |  |  |  |  |  |  |  |  |  |  |  |
| **3.8.12** | JE |  |  |  |  |  |  |  |  |  |  |  |  |  |  |  |  |  |
| **3.8.13** | MMR |  |  |  |  |  |  |  |  |  |  |  |  |  |  |  |  |  |

**3.9 Please record the vaccines received and returned from the session sites under the sub-center (Month September 2016)**

| **Q no** | **Vaccine name** | **Session 1** | | | | **Session 2** | | | | **Session 3** | | | | **Session 4** | | | | |
| --- | --- | --- | --- | --- | --- | --- | --- | --- | --- | --- | --- | --- | --- | --- | --- | --- | --- | --- |
|  |  | **Received vaccines(doses)** | | **Returned vaccine (doses)** | | **Received vaccines(doses)** | | **Returned vaccine (doses)** | | **Received vaccines(doses)** | | **Returned vaccine (doses)** | | **Received vaccines(doses)** | | **Returned vaccine (doses)** | | |
|  |  | Unopen | Open | Unopen | Open | Unopen | Open | Unopen | Open | Unopen | Open | Unopen | Open | Unopen | Open | Unopen | Open |  |
| **3.9.1** | OPV |  |  |  |  |  |  |  |  |  |  |  |  |  |  |  |  |  |
| **3.9.2** | HBV |  |  |  |  |  |  |  |  |  |  |  |  |  |  |  |  |  |
| **3.9.3** | BCG |  |  |  |  |  |  |  |  |  |  |  |  |  |  |  |  |  |
| **3.9.4** | Penta |  |  |  |  |  |  |  |  |  |  |  |  |  |  |  |  |  |
| **3.9.5** | DPT |  |  |  |  |  |  |  |  |  |  |  |  |  |  |  |  |  |
| **3.9.6** | Rota |  |  |  |  |  |  |  |  |  |  |  |  |  |  |  |  |  |
| **3.9.7** | IPV |  |  |  |  |  |  |  |  |  |  |  |  |  |  |  |  |  |
| **3.9.8** | Measles |  |  |  |  |  |  |  |  |  |  |  |  |  |  |  |  |  |
| **3.9.9** | MR |  |  |  |  |  |  |  |  |  |  |  |  |  |  |  |  |  |
| **3.9.10** | TT |  |  |  |  |  |  |  |  |  |  |  |  |  |  |  |  |  |
| **3.9.11** | PCV |  |  |  |  |  |  |  |  |  |  |  |  |  |  |  |  |  |
| **3.9.12** | JE |  |  |  |  |  |  |  |  |  |  |  |  |  |  |  |  |  |
| **3.9.13** | MMR |  |  |  |  |  |  |  |  |  |  |  |  |  |  |  |  |  |

**3.10 Please record the vaccines received and returned from the session sites under the sub-center (Month October 2016)**

| **Q no** | **Vaccine name** | **Session 1** | | | | **Session 2** | | | | **Session 3** | | | | **Session 4** | | | | |
| --- | --- | --- | --- | --- | --- | --- | --- | --- | --- | --- | --- | --- | --- | --- | --- | --- | --- | --- |
|  |  | **Received vaccines(doses)** | | **Returned vaccine (doses)** | | **Received vaccines(doses)** | | **Returned vaccine (doses)** | | **Received vaccines(doses)** | | **Returned vaccine (doses)** | | **Received vaccines(doses)** | | **Returned vaccine (doses)** | | |
|  |  | Unopen | Open | Unopen | Open | Unopen | Open | Unopen | Open | Unopen | Open | Unopen | Open | Unopen | Open | Unopen | Open |  |
| **3.10.1** | OPV |  |  |  |  |  |  |  |  |  |  |  |  |  |  |  |  |  |
| **3.10.2** | HBV |  |  |  |  |  |  |  |  |  |  |  |  |  |  |  |  |  |
| **3.10.3** | BCG |  |  |  |  |  |  |  |  |  |  |  |  |  |  |  |  |  |
| **3.10.4** | Penta |  |  |  |  |  |  |  |  |  |  |  |  |  |  |  |  |  |
| **3.10.5** | DPT |  |  |  |  |  |  |  |  |  |  |  |  |  |  |  |  |  |
| **3.10.6** | Rota |  |  |  |  |  |  |  |  |  |  |  |  |  |  |  |  |  |
| **3.10.7** | IPV |  |  |  |  |  |  |  |  |  |  |  |  |  |  |  |  |  |
| **3.10.8** | Measles |  |  |  |  |  |  |  |  |  |  |  |  |  |  |  |  |  |
| **3.10.9** | MR |  |  |  |  |  |  |  |  |  |  |  |  |  |  |  |  |  |
| **3.10.10** | TT |  |  |  |  |  |  |  |  |  |  |  |  |  |  |  |  |  |
| **3.10.11** | PCV |  |  |  |  |  |  |  |  |  |  |  |  |  |  |  |  |  |
| **3.10.12** | JE |  |  |  |  |  |  |  |  |  |  |  |  |  |  |  |  |  |
| **3.10.13** | MMR |  |  |  |  |  |  |  |  |  |  |  |  |  |  |  |  |  |

**3.11 Please record the vaccines received and returned from the session sites under the sub-center (Month November 2016)**

| **Q no** | **Vaccine name** | **Session 1** | | | | **Session 2** | | | | **Session 3** | | | | **Session 4** | | | | |
| --- | --- | --- | --- | --- | --- | --- | --- | --- | --- | --- | --- | --- | --- | --- | --- | --- | --- | --- |
|  |  | **Received vaccines(doses)** | | **Returned vaccine (doses)** | | **Received vaccines(doses)** | | **Returned vaccine (doses)** | | **Received vaccines(doses)** | | **Returned vaccine (doses)** | | **Received vaccines(doses)** | | **Returned vaccine (doses)** | | |
|  |  | Unopen | Open | Unopen | Open | Unopen | Open | Unopen | Open | Unopen | Open | Unopen | Open | Unopen | Open | Unopen | Open |  |
| **3.11.1** | OPV |  |  |  |  |  |  |  |  |  |  |  |  |  |  |  |  |  |
| **3.11.2** | HBV |  |  |  |  |  |  |  |  |  |  |  |  |  |  |  |  |  |
| **3.11.3** | BCG |  |  |  |  |  |  |  |  |  |  |  |  |  |  |  |  |  |
| **3.11.4** | Penta |  |  |  |  |  |  |  |  |  |  |  |  |  |  |  |  |  |
| **3.11.5** | DPT |  |  |  |  |  |  |  |  |  |  |  |  |  |  |  |  |  |
| **3.11.6** | Rota |  |  |  |  |  |  |  |  |  |  |  |  |  |  |  |  |  |
| **3.11.7** | IPV |  |  |  |  |  |  |  |  |  |  |  |  |  |  |  |  |  |
| **3.11.8** | Measles |  |  |  |  |  |  |  |  |  |  |  |  |  |  |  |  |  |
| **3.11.9** | MR |  |  |  |  |  |  |  |  |  |  |  |  |  |  |  |  |  |
| **3.11.10** | TT |  |  |  |  |  |  |  |  |  |  |  |  |  |  |  |  |  |
| **3.11.11** | PCV |  |  |  |  |  |  |  |  |  |  |  |  |  |  |  |  |  |
| **3.11.12** | JE |  |  |  |  |  |  |  |  |  |  |  |  |  |  |  |  |  |
| **3.11.13** | MMR |  |  |  |  |  |  |  |  |  |  |  |  |  |  |  |  |  |

**3.12 Please record the vaccines received and returned from the session sites under the sub-center (Month December 2016)**

| **Q no** | **Vaccine name** | **Session 1** | | | | **Session 2** | | | | **Session 3** | | | | **Session 4** | | | |
| --- | --- | --- | --- | --- | --- | --- | --- | --- | --- | --- | --- | --- | --- | --- | --- | --- | --- |
|  |  | **Received vaccines(doses)** | | **Returned vaccine (doses)** | | **Received vaccines(doses)** | | **Returned vaccine (doses)** | | **Received vaccines(doses)** | | **Returned vaccine (doses)** | | **Received vaccines(doses)** | | **Returned vaccine (doses)** | |
|  |  | Unopen | Open | Unopen | Open | Unopen | Open | Unopen | Open | Unopen | Open | Unopen | Open | Unopen | Open | Unopen | Open |
| **3.12.1** | OPV |  |  |  |  |  |  |  |  |  |  |  |  |  |  |  |  |
| **3.12.2** | HBV |  |  |  |  |  |  |  |  |  |  |  |  |  |  |  |  |
| **3.12.3** | BCG |  |  |  |  |  |  |  |  |  |  |  |  |  |  |  |  |
| **3.12.4** | Penta |  |  |  |  |  |  |  |  |  |  |  |  |  |  |  |  |
| **3.12.5** | DPT |  |  |  |  |  |  |  |  |  |  |  |  |  |  |  |  |
| **3.12.6** | Rota |  |  |  |  |  |  |  |  |  |  |  |  |  |  |  |  |
| **3.12.7** | IPV |  |  |  |  |  |  |  |  |  |  |  |  |  |  |  |  |
| **3.12.8** | Measles |  |  |  |  |  |  |  |  |  |  |  |  |  |  |  |  |
| **3.12.9** | MR |  |  |  |  |  |  |  |  |  |  |  |  |  |  |  |  |
| **3.12.10** | TT |  |  |  |  |  |  |  |  |  |  |  |  |  |  |  |  |
| **3.12.11** | PCV |  |  |  |  |  |  |  |  |  |  |  |  |  |  |  |  |
| **3.12.12** | JE |  |  |  |  |  |  |  |  |  |  |  |  |  |  |  |  |
| **3.12.13** | MMR |  |  |  |  |  |  |  |  |  |  |  |  |  |  |  |  |

**3.13 Please record the vaccines received and returned from the session sites under the sub-center (Month January 2017)**

| **Q no** | **Vaccine name** | **Session 1** | | | | **Session 2** | | | | **Session 3** | | | | **Session 4** | | | | |
| --- | --- | --- | --- | --- | --- | --- | --- | --- | --- | --- | --- | --- | --- | --- | --- | --- | --- | --- |
|  |  | **Received vaccines(doses)** | | **Returned vaccine (doses)** | | **Received vaccines(doses)** | | **Returned vaccine (doses)** | | **Received vaccines(doses)** | | **Returned vaccine (doses)** | | **Received vaccines(doses)** | | **Returned vaccine (doses)** | | |
|  |  | Unopen | Open | Unopen | Open | Unopen | Open | Unopen | Open | Unopen | Open | Unopen | Open | Unopen | Open | Unopen | Open |  |
| **3.13.1** | OPV |  |  |  |  |  |  |  |  |  |  |  |  |  |  |  |  |  |
| **3.13.2** | HBV |  |  |  |  |  |  |  |  |  |  |  |  |  |  |  |  |  |
| **3.13.3** | BCG |  |  |  |  |  |  |  |  |  |  |  |  |  |  |  |  |  |
| **3.13.4** | Penta |  |  |  |  |  |  |  |  |  |  |  |  |  |  |  |  |  |
| **3.13.5** | DPT |  |  |  |  |  |  |  |  |  |  |  |  |  |  |  |  |  |
| **3.13.6** | Rota |  |  |  |  |  |  |  |  |  |  |  |  |  |  |  |  |  |
| **3.13.7** | IPV |  |  |  |  |  |  |  |  |  |  |  |  |  |  |  |  |  |
| **3.13.8** | Measles |  |  |  |  |  |  |  |  |  |  |  |  |  |  |  |  |  |
| **3.13.9** | MR |  |  |  |  |  |  |  |  |  |  |  |  |  |  |  |  |  |
| **3.13.10** | TT |  |  |  |  |  |  |  |  |  |  |  |  |  |  |  |  |  |
| **3.13.11** | PCV |  |  |  |  |  |  |  |  |  |  |  |  |  |  |  |  |  |
| **3.13.12** | JE |  |  |  |  |  |  |  |  |  |  |  |  |  |  |  |  |  |
| **3.13.13** | MMR |  |  |  |  |  |  |  |  |  |  |  |  |  |  |  |  |  |

**3.14 Please record the vaccines received and returned from the session sites under the sub-center (Month February 2017)**

| **Q no** | **Vaccine name** | **Session 1** | | | | **Session 2** | | | | **Session 3** | | | | **Session 4** | | | |
| --- | --- | --- | --- | --- | --- | --- | --- | --- | --- | --- | --- | --- | --- | --- | --- | --- | --- |
|  |  | **Received vaccines(doses)** | | **Returned vaccine (doses)** | | **Received vaccines(doses)** | | **Returned vaccine (doses)** | | **Received vaccines(doses)** | | **Returned vaccine (doses)** | | **Received vaccines(doses)** | | **Returned vaccine (doses)** | |
|  |  | Unopen | Open | Unopen | Open | Unopen | Open | Unopen | Open | Unopen | Open | Unopen | Open | Unopen | Open | Unopen | Open |
| **3.14.1** | OPV |  |  |  |  |  |  |  |  |  |  |  |  |  |  |  |  |
| **3.14.2** | HBV |  |  |  |  |  |  |  |  |  |  |  |  |  |  |  |  |
| **3.14.3** | BCG |  |  |  |  |  |  |  |  |  |  |  |  |  |  |  |  |
| **3.14.4** | Penta |  |  |  |  |  |  |  |  |  |  |  |  |  |  |  |  |
| **3.14.5** | DPT |  |  |  |  |  |  |  |  |  |  |  |  |  |  |  |  |
| **3.14.6** | Rota |  |  |  |  |  |  |  |  |  |  |  |  |  |  |  |  |
| **3.14.7** | IPV |  |  |  |  |  |  |  |  |  |  |  |  |  |  |  |  |
| **3.14.8** | Measles |  |  |  |  |  |  |  |  |  |  |  |  |  |  |  |  |
| **3.14.9** | MR |  |  |  |  |  |  |  |  |  |  |  |  |  |  |  |  |
| **3.14.10** | TT |  |  |  |  |  |  |  |  |  |  |  |  |  |  |  |  |
| **3.14.11** | PCV |  |  |  |  |  |  |  |  |  |  |  |  |  |  |  |  |
| **3.14.12** | JE |  |  |  |  |  |  |  |  |  |  |  |  |  |  |  |  |
| **3.14.13** | MMR |  |  |  |  |  |  |  |  |  |  |  |  |  |  |  |  |

**3.15 Please record the vaccines received and returned from the session sites under the sub-center (Month March 2017)**

| **Q no** | **Vaccine name** | **Session 1** | | | | **Session 2** | | | | **Session 3** | | | | **Session 4** | | | | |
| --- | --- | --- | --- | --- | --- | --- | --- | --- | --- | --- | --- | --- | --- | --- | --- | --- | --- | --- |
|  |  | **Received vaccines(doses)** | | **Returned vaccine (doses)** | | **Received vaccines(doses)** | | **Returned vaccine (doses)** | | **Received vaccines(doses)** | | **Returned vaccine (doses)** | | **Received vaccines(doses)** | | **Returned vaccine (doses)** | | |
|  |  | Unopen | Open | Unopen | Open | Unopen | Open | Unopen | Open | Unopen | Open | Unopen | Open | Unopen | Open | Unopen | Open |  |
| **3.15.1** | OPV |  |  |  |  |  |  |  |  |  |  |  |  |  |  |  |  |  |
| **3.15.2** | HBV |  |  |  |  |  |  |  |  |  |  |  |  |  |  |  |  |  |
| **3.15.3** | BCG |  |  |  |  |  |  |  |  |  |  |  |  |  |  |  |  |  |
| **3.15.4** | Penta |  |  |  |  |  |  |  |  |  |  |  |  |  |  |  |  |  |
| **3.15.5** | DPT |  |  |  |  |  |  |  |  |  |  |  |  |  |  |  |  |  |
| **3.15.6** | Rota |  |  |  |  |  |  |  |  |  |  |  |  |  |  |  |  |  |
| **3.15.7** | IPV |  |  |  |  |  |  |  |  |  |  |  |  |  |  |  |  |  |
| **3.15.8** | Measles |  |  |  |  |  |  |  |  |  |  |  |  |  |  |  |  |  |
| **3.15.9** | MR |  |  |  |  |  |  |  |  |  |  |  |  |  |  |  |  |  |
| **3.15.10** | TT |  |  |  |  |  |  |  |  |  |  |  |  |  |  |  |  |  |
| **3.15.11** | PCV |  |  |  |  |  |  |  |  |  |  |  |  |  |  |  |  |  |
| **3.15.12** | JE |  |  |  |  |  |  |  |  |  |  |  |  |  |  |  |  |  |
| **3.15.13** | MMR |  |  |  |  |  |  |  |  |  |  |  |  |  |  |  |  |  |

**3.16 Please record the vaccines received and returned from the session sites under the sub-center (Month April 2017)**

| **Q no** | **Vaccine name** | **Session 1** | | | | **Session 2** | | | | **Session 3** | | | | **Session 4** | | | | |
| --- | --- | --- | --- | --- | --- | --- | --- | --- | --- | --- | --- | --- | --- | --- | --- | --- | --- | --- |
|  |  | **Received vaccines(doses)** | | **Returned vaccine (doses)** | | **Received vaccines(doses)** | | **Returned vaccine (doses)** | | **Received vaccines(doses)** | | **Returned vaccine (doses)** | | **Received vaccines(doses)** | | **Returned vaccine (doses)** | | |
|  |  | Unopen | Open | Unopen | Open | Unopen | Open | Unopen | Open | Unopen | Open | Unopen | Open | Unopen | Open | Unopen | Open |  |
| **3.16.1** | OPV |  |  |  |  |  |  |  |  |  |  |  |  |  |  |  |  |  |
| **3.16.2** | HBV |  |  |  |  |  |  |  |  |  |  |  |  |  |  |  |  |  |
| **3.16.3** | BCG |  |  |  |  |  |  |  |  |  |  |  |  |  |  |  |  |  |
| **3.16.4** | Penta |  |  |  |  |  |  |  |  |  |  |  |  |  |  |  |  |  |
| **3.16.5** | DPT |  |  |  |  |  |  |  |  |  |  |  |  |  |  |  |  |  |
| **3.16.6** | Rota |  |  |  |  |  |  |  |  |  |  |  |  |  |  |  |  |  |
| **3.16.7** | IPV |  |  |  |  |  |  |  |  |  |  |  |  |  |  |  |  |  |
| **3.16.8** | Measles |  |  |  |  |  |  |  |  |  |  |  |  |  |  |  |  |  |
| **3.16.9** | MR |  |  |  |  |  |  |  |  |  |  |  |  |  |  |  |  |  |
| **3.16.10** | TT |  |  |  |  |  |  |  |  |  |  |  |  |  |  |  |  |  |
| **3.16.11** | PCV |  |  |  |  |  |  |  |  |  |  |  |  |  |  |  |  |  |
| **3.16.12** | JE |  |  |  |  |  |  |  |  |  |  |  |  |  |  |  |  |  |
| **3.16.13** | MMR |  |  |  |  |  |  |  |  |  |  |  |  |  |  |  |  |  |

**3.17 Please record the vaccines received and returned from the session sites under the sub-center (Month May 2017)**

| **Q no** | **Vaccine name** | **Session 1** | | | | **Session 2** | | | | **Session 3** | | | | **Session 4** | | | | |
| --- | --- | --- | --- | --- | --- | --- | --- | --- | --- | --- | --- | --- | --- | --- | --- | --- | --- | --- |
|  |  | **Received vaccines(doses)** | | **Returned vaccine (doses)** | | **Received vaccines(doses)** | | **Returned vaccine (doses)** | | **Received vaccines(doses)** | | **Returned vaccine (doses)** | | **Received vaccines(doses)** | | **Returned vaccine (doses)** | | |
|  |  | Unopen | Open | Unopen | Open | Unopen | Open | Unopen | Open | Unopen | Open | Unopen | Open | Unopen | Open | Unopen | Open |  |
| **3.17.1** | OPV |  |  |  |  |  |  |  |  |  |  |  |  |  |  |  |  |  |
| **3.17.2** | HBV |  |  |  |  |  |  |  |  |  |  |  |  |  |  |  |  |  |
| **3.17.3** | BCG |  |  |  |  |  |  |  |  |  |  |  |  |  |  |  |  |  |
| **3.17.4** | Penta |  |  |  |  |  |  |  |  |  |  |  |  |  |  |  |  |  |
| **3.17.5** | DPT |  |  |  |  |  |  |  |  |  |  |  |  |  |  |  |  |  |
| **3.17.6** | Rota |  |  |  |  |  |  |  |  |  |  |  |  |  |  |  |  |  |
| **3.17.7** | IPV |  |  |  |  |  |  |  |  |  |  |  |  |  |  |  |  |  |
| **3.17.8** | Measles |  |  |  |  |  |  |  |  |  |  |  |  |  |  |  |  |  |
| **3.17.9** | MR |  |  |  |  |  |  |  |  |  |  |  |  |  |  |  |  |  |
| **3.17.10** | TT |  |  |  |  |  |  |  |  |  |  |  |  |  |  |  |  |  |
| **3.17.11** | PCV |  |  |  |  |  |  |  |  |  |  |  |  |  |  |  |  |  |
| **3.17.12** | JE |  |  |  |  |  |  |  |  |  |  |  |  |  |  |  |  |  |
| **3.17.13** | MMR |  |  |  |  |  |  |  |  |  |  |  |  |  |  |  |  |  |

**3.18 Please record the vaccines received and returned from the session sites under the sub-center (Month Jun 2017)**

| **Q no** | **Vaccine name** | **Session 1** | | | | **Session 2** | | | | **Session 3** | | | | **Session 4** | | | | |
| --- | --- | --- | --- | --- | --- | --- | --- | --- | --- | --- | --- | --- | --- | --- | --- | --- | --- | --- |
|  |  | **Received vaccines(doses)** | | **Returned vaccine (doses)** | | **Received vaccines(doses)** | | **Returned vaccine (doses)** | | **Received vaccines(doses)** | | **Returned vaccine (doses)** | | **Received vaccines(doses)** | | **Returned vaccine (doses)** | | |
|  |  | Unopen | Open | Unopen | Open | Unopen | Open | Unopen | Open | Unopen | Open | Unopen | Open | Unopen | Open | Unopen | Open |  |
| **3.18.1** | OPV |  |  |  |  |  |  |  |  |  |  |  |  |  |  |  |  |  |
| **3.18.2** | HBV |  |  |  |  |  |  |  |  |  |  |  |  |  |  |  |  |  |
| **3.18.3** | BCG |  |  |  |  |  |  |  |  |  |  |  |  |  |  |  |  |  |
| **3.18.4** | Penta |  |  |  |  |  |  |  |  |  |  |  |  |  |  |  |  |  |
| **3.18.5** | DPT |  |  |  |  |  |  |  |  |  |  |  |  |  |  |  |  |  |
| **3.18.6** | Rota |  |  |  |  |  |  |  |  |  |  |  |  |  |  |  |  |  |
| **3.18.7** | IPV |  |  |  |  |  |  |  |  |  |  |  |  |  |  |  |  |  |
| **3.18.8** | Measles |  |  |  |  |  |  |  |  |  |  |  |  |  |  |  |  |  |
| **3.18.9** | MR |  |  |  |  |  |  |  |  |  |  |  |  |  |  |  |  |  |
| **3.18.10** | TT |  |  |  |  |  |  |  |  |  |  |  |  |  |  |  |  |  |
| **3.18.11** | PCV |  |  |  |  |  |  |  |  |  |  |  |  |  |  |  |  |  |
| **3.18.12** | JE |  |  |  |  |  |  |  |  |  |  |  |  |  |  |  |  |  |
| **3.18.13** | MMR |  |  |  |  |  |  |  |  |  |  |  |  |  |  |  |  |  |

**3.19 Please record the vaccines received and returned from the session sites under the sub-center (Month July 2017)**

| **Q no** | **Vaccine name** | **Session 1** | | | | **Session 2** | | | | **Session 3** | | | | **Session 4** | | | |
| --- | --- | --- | --- | --- | --- | --- | --- | --- | --- | --- | --- | --- | --- | --- | --- | --- | --- |
|  |  | **Received vaccines(doses)** | | **Returned vaccine (doses)** | | **Received vaccines(doses)** | | **Returned vaccine (doses)** | | **Received vaccines(doses)** | | **Returned vaccine (doses)** | | **Received vaccines(doses)** | | **Returned vaccine (doses)** | |
|  |  | Unopen | Open | Unopen | Open | Unopen | Open | Unopen | Open | Unopen | Open | Unopen | Open | Unopen | Open | Unopen | Open |
| **3.19.1** | OPV |  |  |  |  |  |  |  |  |  |  |  |  |  |  |  |  |
| **3.19.2** | HBV |  |  |  |  |  |  |  |  |  |  |  |  |  |  |  |  |
| **3.19.3** | BCG |  |  |  |  |  |  |  |  |  |  |  |  |  |  |  |  |
| **3.19.4** | Penta |  |  |  |  |  |  |  |  |  |  |  |  |  |  |  |  |
| **3.19.5** | DPT |  |  |  |  |  |  |  |  |  |  |  |  |  |  |  |  |
| **3.19.6** | Rota |  |  |  |  |  |  |  |  |  |  |  |  |  |  |  |  |
| **3.19.7** | IPV |  |  |  |  |  |  |  |  |  |  |  |  |  |  |  |  |
| **3.19.8** | Measles |  |  |  |  |  |  |  |  |  |  |  |  |  |  |  |  |
| **3.19.9** | MR |  |  |  |  |  |  |  |  |  |  |  |  |  |  |  |  |
| **3.19.10** | TT |  |  |  |  |  |  |  |  |  |  |  |  |  |  |  |  |
| **3.19.11** | PCV |  |  |  |  |  |  |  |  |  |  |  |  |  |  |  |  |
| **3.19.12** | JE |  |  |  |  |  |  |  |  |  |  |  |  |  |  |  |  |
| **3.19.13** | MMR |  |  |  |  |  |  |  |  |  |  |  |  |  |  |  |  |

**3.20 Please record the vaccines received and returned from the session sites under the sub-center (Month August 2017)**

| **Q no** | **Vaccine name** | **Session 1** | | | | **Session 2** | | | | **Session 3** | | | | **Session 4** | | | | |
| --- | --- | --- | --- | --- | --- | --- | --- | --- | --- | --- | --- | --- | --- | --- | --- | --- | --- | --- |
|  |  | **Received vaccines(doses)** | | **Returned vaccine (doses)** | | **Received vaccines(doses)** | | **Returned vaccine (doses)** | | **Received vaccines(doses)** | | **Returned vaccine (doses)** | | **Received vaccines(doses)** | | **Returned vaccine (doses)** | | |
|  |  | Unopen | Open | Unopen | Open | Unopen | Open | Unopen | Open | Unopen | Open | Unopen | Open | Unopen | Open | Unopen | Open |  |
| **3.20.1** | OPV |  |  |  |  |  |  |  |  |  |  |  |  |  |  |  |  |  |
| **3.20.2** | HBV |  |  |  |  |  |  |  |  |  |  |  |  |  |  |  |  |  |
| **3.20.3** | BCG |  |  |  |  |  |  |  |  |  |  |  |  |  |  |  |  |  |
| **3.20.4** | Penta |  |  |  |  |  |  |  |  |  |  |  |  |  |  |  |  |  |
| **3.20.5** | DPT |  |  |  |  |  |  |  |  |  |  |  |  |  |  |  |  |  |
| **3.20.6** | Rota |  |  |  |  |  |  |  |  |  |  |  |  |  |  |  |  |  |
| **3.20.7** | IPV |  |  |  |  |  |  |  |  |  |  |  |  |  |  |  |  |  |
| **3.20.8** | Measles |  |  |  |  |  |  |  |  |  |  |  |  |  |  |  |  |  |
| **3.20.9** | MR |  |  |  |  |  |  |  |  |  |  |  |  |  |  |  |  |  |
| **3.20.10** | TT |  |  |  |  |  |  |  |  |  |  |  |  |  |  |  |  |  |
| **3.20.11** | PCV |  |  |  |  |  |  |  |  |  |  |  |  |  |  |  |  |  |
| **3.20.12** | JE |  |  |  |  |  |  |  |  |  |  |  |  |  |  |  |  |  |
| **3.20.13** | MMR |  |  |  |  |  |  |  |  |  |  |  |  |  |  |  |  |  |

**3.21 Please record the vaccines received and returned from the session sites under the sub-center (Month September 2017)**

| **Q no** | **Vaccine name** | **Session 1** | | | | **Session 2** | | | | **Session 3** | | | | **Session 4** | | | | |
| --- | --- | --- | --- | --- | --- | --- | --- | --- | --- | --- | --- | --- | --- | --- | --- | --- | --- | --- |
|  |  | **Received vaccines(doses)** | | **Returned vaccine (doses)** | | **Received vaccines(doses)** | | **Returned vaccine (doses)** | | **Received vaccines(doses)** | | **Returned vaccine (doses)** | | **Received vaccines(doses)** | | **Returned vaccine (doses)** | | |
|  |  | Unopen | Open | Unopen | Open | Unopen | Open | Unopen | Open | Unopen | Open | Unopen | Open | Unopen | Open | Unopen | Open |  |
| **3.21.1** | OPV |  |  |  |  |  |  |  |  |  |  |  |  |  |  |  |  |  |
| **3.21.2** | HBV |  |  |  |  |  |  |  |  |  |  |  |  |  |  |  |  |  |
| **3.21.3** | BCG |  |  |  |  |  |  |  |  |  |  |  |  |  |  |  |  |  |
| **3.21.4** | Penta |  |  |  |  |  |  |  |  |  |  |  |  |  |  |  |  |  |
| **3.21.5** | DPT |  |  |  |  |  |  |  |  |  |  |  |  |  |  |  |  |  |
| **3.21.6** | Rota |  |  |  |  |  |  |  |  |  |  |  |  |  |  |  |  |  |
| **3.21.7** | IPV |  |  |  |  |  |  |  |  |  |  |  |  |  |  |  |  |  |
| **3.21.8** | Measles |  |  |  |  |  |  |  |  |  |  |  |  |  |  |  |  |  |
| **3.21.9** | MR |  |  |  |  |  |  |  |  |  |  |  |  |  |  |  |  |  |
| **3.21.10** | TT |  |  |  |  |  |  |  |  |  |  |  |  |  |  |  |  |  |
| **3.21.11** | PCV |  |  |  |  |  |  |  |  |  |  |  |  |  |  |  |  |  |
| **3.21.12** | JE |  |  |  |  |  |  |  |  |  |  |  |  |  |  |  |  |  |
| **3.21.13** | MMR |  |  |  |  |  |  |  |  |  |  |  |  |  |  |  |  |  |

**3.22 Please record the vaccines received and returned from the session sites under the sub-center (Month October 2017)**

| **Q no** | **Vaccine name** | **Session 1** | | | | **Session 2** | | | | **Session 3** | | | | **Session 4** | | | | |
| --- | --- | --- | --- | --- | --- | --- | --- | --- | --- | --- | --- | --- | --- | --- | --- | --- | --- | --- |
|  |  | **Received vaccines(doses)** | | **Returned vaccine (doses)** | | **Received vaccines(doses)** | | **Returned vaccine (doses)** | | **Received vaccines(doses)** | | **Returned vaccine (doses)** | | **Received vaccines(doses)** | | **Returned vaccine (doses)** | | |
|  |  | Unopen | Open | Unopen | Open | Unopen | Open | Unopen | Open | Unopen | Open | Unopen | Open | Unopen | Open | Unopen | Open |  |
| **3.22.1** | OPV |  |  |  |  |  |  |  |  |  |  |  |  |  |  |  |  |  |
| **3.22.2** | HBV |  |  |  |  |  |  |  |  |  |  |  |  |  |  |  |  |  |
| **3.22.3** | BCG |  |  |  |  |  |  |  |  |  |  |  |  |  |  |  |  |  |
| **3.22.4** | Penta |  |  |  |  |  |  |  |  |  |  |  |  |  |  |  |  |  |
| **3.22.5** | DPT |  |  |  |  |  |  |  |  |  |  |  |  |  |  |  |  |  |
| **3.22.6** | Rota |  |  |  |  |  |  |  |  |  |  |  |  |  |  |  |  |  |
| **3.22.7** | IPV |  |  |  |  |  |  |  |  |  |  |  |  |  |  |  |  |  |
| **3.22.8** | Measles |  |  |  |  |  |  |  |  |  |  |  |  |  |  |  |  |  |
| **3.22.9** | MR |  |  |  |  |  |  |  |  |  |  |  |  |  |  |  |  |  |
| **3.22.10** | TT |  |  |  |  |  |  |  |  |  |  |  |  |  |  |  |  |  |
| **3.22.11** | PCV |  |  |  |  |  |  |  |  |  |  |  |  |  |  |  |  |  |
| **3.22.12** | JE |  |  |  |  |  |  |  |  |  |  |  |  |  |  |  |  |  |
| **3.22.13** | MMR |  |  |  |  |  |  |  |  |  |  |  |  |  |  |  |  |  |

**3.23 Please record the vaccines received and returned from the session sites under the sub-center (Month November 2017)**

| **Q no** | **Vaccine name** | **Session 1** | | | | **Session 2** | | | | **Session 3** | | | | **Session 4** | | | |
| --- | --- | --- | --- | --- | --- | --- | --- | --- | --- | --- | --- | --- | --- | --- | --- | --- | --- |
|  |  | **Received vaccines(doses)** | | **Returned vaccine (doses)** | | **Received vaccines(doses)** | | **Returned vaccine (doses)** | | **Received vaccines(doses)** | | **Returned vaccine (doses)** | | **Received vaccines(doses)** | | **Returned vaccine (doses)** | |
|  |  | Unopen | Open | Unopen | Open | Unopen | Open | Unopen | Open | Unopen | Open | Unopen | Open | Unopen | Open | Unopen | Open |
| **3.23.1** | OPV |  |  |  |  |  |  |  |  |  |  |  |  |  |  |  |  |
| **3.23.2** | HBV |  |  |  |  |  |  |  |  |  |  |  |  |  |  |  |  |
| **3.23.3** | BCG |  |  |  |  |  |  |  |  |  |  |  |  |  |  |  |  |
| **3.23.4** | Penta |  |  |  |  |  |  |  |  |  |  |  |  |  |  |  |  |
| **3.23.5** | DPT |  |  |  |  |  |  |  |  |  |  |  |  |  |  |  |  |
| **3.23.6** | Rota |  |  |  |  |  |  |  |  |  |  |  |  |  |  |  |  |
| **3.23.7** | IPV |  |  |  |  |  |  |  |  |  |  |  |  |  |  |  |  |
| **3.23.8** | Measles |  |  |  |  |  |  |  |  |  |  |  |  |  |  |  |  |
| **3.23.9** | MR |  |  |  |  |  |  |  |  |  |  |  |  |  |  |  |  |
| **3.23.10** | TT |  |  |  |  |  |  |  |  |  |  |  |  |  |  |  |  |
| **3.23.11** | PCV |  |  |  |  |  |  |  |  |  |  |  |  |  |  |  |  |
| **3.23.12** | JE |  |  |  |  |  |  |  |  |  |  |  |  |  |  |  |  |
| **3.23.13** | MMR |  |  |  |  |  |  |  |  |  |  |  |  |  |  |  |  |

**3.24 Please record the vaccines received and returned from the session sites under the sub-center (Month December 2017)**

| **Q no** | **Vaccine name** | **Session 1** | | | | **Session 2** | | | | **Session 3** | | | | **Session 4** | | | | |
| --- | --- | --- | --- | --- | --- | --- | --- | --- | --- | --- | --- | --- | --- | --- | --- | --- | --- | --- |
|  |  | **Received vaccines(doses)** | | **Returned vaccine (doses)** | | **Received vaccines(doses)** | | **Returned vaccine (doses)** | | **Received vaccines(doses)** | | **Returned vaccine (doses)** | | **Received vaccines(doses)** | | **Returned vaccine (doses)** | | |
|  |  | Unopen | Open | Unopen | Open | Unopen | Open | Unopen | Open | Unopen | Open | Unopen | Open | Unopen | Open | Unopen | Open |  |
| **3.24.1** | OPV |  |  |  |  |  |  |  |  |  |  |  |  |  |  |  |  |  |
| **3.24.2** | HBV |  |  |  |  |  |  |  |  |  |  |  |  |  |  |  |  |  |
| **3.24.3** | BCG |  |  |  |  |  |  |  |  |  |  |  |  |  |  |  |  |  |
| **3.24.4** | Penta |  |  |  |  |  |  |  |  |  |  |  |  |  |  |  |  |  |
| **3.24.5** | DPT |  |  |  |  |  |  |  |  |  |  |  |  |  |  |  |  |  |
| **3.24.6** | Rota |  |  |  |  |  |  |  |  |  |  |  |  |  |  |  |  |  |
| **3.24.7** | IPV |  |  |  |  |  |  |  |  |  |  |  |  |  |  |  |  |  |
| **3.24.8** | Measles |  |  |  |  |  |  |  |  |  |  |  |  |  |  |  |  |  |
| **3.24.9** | MR |  |  |  |  |  |  |  |  |  |  |  |  |  |  |  |  |  |
| **3.24.10** | TT |  |  |  |  |  |  |  |  |  |  |  |  |  |  |  |  |  |
| **3.24.11** | PCV |  |  |  |  |  |  |  |  |  |  |  |  |  |  |  |  |  |
| **3.24.12** | JE |  |  |  |  |  |  |  |  |  |  |  |  |  |  |  |  |  |
| **3.24.13** | MMR |  |  |  |  |  |  |  |  |  |  |  |  |  |  |  |  |  |

**Section C: Vaccination coverage information**

**4.1 Please record the number of beneficiaries received vaccines at the session sites for the month January 2016**

|  |  | **Session 1** | | **Session 2** | | **Session 3** | | **Session 4** | | **Session 5** | |
| --- | --- | --- | --- | --- | --- | --- | --- | --- | --- | --- | --- |
|  |  | Scheduled doses | Actual given doses | Scheduled doses | Actual given doses | Scheduled doses | Actual given doses | Scheduled doses | Actual given doses | Scheduled doses | Actual given doses |
| **4.1.1** | OPV |  |  |  |  |  |  |  |  |  |  |
| **4.1.2** | HBV |  |  |  |  |  |  |  |  |  |  |
| **4.1.3** | BCG |  |  |  |  |  |  |  |  |  |  |
| **4.1.4** | Pentavalent |  |  |  |  |  |  |  |  |  |  |
| **4.1.5** | DPT |  |  |  |  |  |  |  |  |  |  |
| **4.1.6** | Rotavirus |  |  |  |  |  |  |  |  |  |  |
| **4.1.7** | IPV |  |  |  |  |  |  |  |  |  |  |
| **4.1.8** | Measles |  |  |  |  |  |  |  |  |  |  |
| **4.1.9** | MR |  |  |  |  |  |  |  |  |  |  |
| **4.1.10** | TT |  |  |  |  |  |  |  |  |  |  |
| **4.1.11** | PCV |  |  |  |  |  |  |  |  |  |  |
| **4.1.12** | JE |  |  |  |  |  |  |  |  |  |  |
| **4.1.13** | MMR |  |  |  |  |  |  |  |  |  |  |

**4.2 Please record the number of beneficiaries received vaccines at the session sites for the month February 2016**

|  |  | **Session 1** | | **Session 2** | | **Session 3** | | **Session 4** | | **Session 5** | |
| --- | --- | --- | --- | --- | --- | --- | --- | --- | --- | --- | --- |
|  |  | Scheduled doses | Actual given doses | Scheduled doses | Actual given doses | Scheduled doses | Actual given doses | Scheduled doses | Actual given doses | Scheduled doses | Actual given doses |
| **4.2.1** | OPV |  |  |  |  |  |  |  |  |  |  |
| **4.2.2** | HBV |  |  |  |  |  |  |  |  |  |  |
| **4.2.3** | BCG |  |  |  |  |  |  |  |  |  |  |
| **4.2.4** | Pentavalent |  |  |  |  |  |  |  |  |  |  |
| **4.2.5** | DPT |  |  |  |  |  |  |  |  |  |  |
| **4.2.6** | Rotavirus |  |  |  |  |  |  |  |  |  |  |
| **4.2.7** | IPV |  |  |  |  |  |  |  |  |  |  |
| **4.2.8** | Measles |  |  |  |  |  |  |  |  |  |  |
| **4.2.9** | MR |  |  |  |  |  |  |  |  |  |  |
| **4.2.10** | TT |  |  |  |  |  |  |  |  |  |  |
| **4.2.11** | PCV |  |  |  |  |  |  |  |  |  |  |
| **4.2.12** | JE |  |  |  |  |  |  |  |  |  |  |
| **4.2.13** | MMR |  |  |  |  |  |  |  |  |  |  |

**4.3 Please record the number of beneficiaries received vaccines at the session sites for the month March 2016**

|  |  | **Session 1** | | **Session 2** | | **Session 3** | | **Session 4** | | **Session 5** | |
| --- | --- | --- | --- | --- | --- | --- | --- | --- | --- | --- | --- |
|  |  | Scheduled doses | Actual given doses | Scheduled doses | Actual given doses | Scheduled doses | Actual given doses | Scheduled doses | Actual given doses | Scheduled doses | Actual given doses |
| **4.3.1** | OPV |  |  |  |  |  |  |  |  |  |  |
| **4.3.2** | HBV |  |  |  |  |  |  |  |  |  |  |
| **4.3.3** | BCG |  |  |  |  |  |  |  |  |  |  |
| **4.3.4** | Pentavalent |  |  |  |  |  |  |  |  |  |  |
| **4.3.5** | DPT |  |  |  |  |  |  |  |  |  |  |
| **4.3.6** | Rotavirus |  |  |  |  |  |  |  |  |  |  |
| **4.3.7** | IPV |  |  |  |  |  |  |  |  |  |  |
| **4.3.8** | Measles |  |  |  |  |  |  |  |  |  |  |
| **4.3.9** | MR |  |  |  |  |  |  |  |  |  |  |
| **4.3.10** | TT |  |  |  |  |  |  |  |  |  |  |
| **4.3.11** | PCV |  |  |  |  |  |  |  |  |  |  |
| **4.3.12** | JE |  |  |  |  |  |  |  |  |  |  |
| **4.3.13** | MMR |  |  |  |  |  |  |  |  |  |  |

**4.4 Please record the number of beneficiaries received vaccines at the session sites for the month April 2016**

|  |  | **Session 1** | | **Session 2** | | **Session 3** | | **Session 4** | | **Session 5** | |
| --- | --- | --- | --- | --- | --- | --- | --- | --- | --- | --- | --- |
|  |  | Scheduled doses | Actual given doses | Scheduled doses | Actual given doses | Scheduled doses | Actual given doses | Scheduled doses | Actual given doses | Scheduled doses | Actual given doses |
| **4.4.1** | OPV |  |  |  |  |  |  |  |  |  |  |
| **4.4.2** | HBV |  |  |  |  |  |  |  |  |  |  |
| **4.4.3** | BCG |  |  |  |  |  |  |  |  |  |  |
| **4.4.4** | Pentavalent |  |  |  |  |  |  |  |  |  |  |
| **4.4.5** | DPT |  |  |  |  |  |  |  |  |  |  |
| **4.4.6** | Rotavirus |  |  |  |  |  |  |  |  |  |  |
| **4.4.7** | IPV |  |  |  |  |  |  |  |  |  |  |
| **4.4.8** | Measles |  |  |  |  |  |  |  |  |  |  |
| **4.4.9** | MR |  |  |  |  |  |  |  |  |  |  |
| **4.4.10** | TT |  |  |  |  |  |  |  |  |  |  |
| **4.4.11** | PCV |  |  |  |  |  |  |  |  |  |  |
| **4.4.12** | JE |  |  |  |  |  |  |  |  |  |  |
| **4.4.13** | MMR |  |  |  |  |  |  |  |  |  |  |

**4.5 Please record the number of beneficiaries received vaccines at the session sites for the month May 2016**

|  |  | **Session 1** | | **Session 2** | | **Session 3** | | **Session 4** | | **Session 5** | |
| --- | --- | --- | --- | --- | --- | --- | --- | --- | --- | --- | --- |
|  |  | Scheduled doses | Actual given doses | Scheduled doses | Actual given doses | Scheduled doses | Actual given doses | Scheduled doses | Actual given doses | Scheduled doses | Actual given doses |
| **4.5.1** | OPV |  |  |  |  |  |  |  |  |  |  |
| **4.5.2** | HBV |  |  |  |  |  |  |  |  |  |  |
| **4.5.3** | BCG |  |  |  |  |  |  |  |  |  |  |
| **4.5.4** | Pentavalent |  |  |  |  |  |  |  |  |  |  |
| **4.5.5** | DPT |  |  |  |  |  |  |  |  |  |  |
| **4.5.6** | Rotavirus |  |  |  |  |  |  |  |  |  |  |
| **4.5.7** | IPV |  |  |  |  |  |  |  |  |  |  |
| **4.5.8** | Measles |  |  |  |  |  |  |  |  |  |  |
| **4.5.9** | MR |  |  |  |  |  |  |  |  |  |  |
| **4.5.10** | TT |  |  |  |  |  |  |  |  |  |  |
| **4.5.11** | PCV |  |  |  |  |  |  |  |  |  |  |
| **4.5.12** | JE |  |  |  |  |  |  |  |  |  |  |
| **4.5.13** | MMR |  |  |  |  |  |  |  |  |  |  |

**4.6 Please record the number of beneficiaries received vaccines at the session sites for the month June 2016**

|  |  | **Session 1** | | **Session 2** | | **Session 3** | | **Session 4** | | **Session 5** | |
| --- | --- | --- | --- | --- | --- | --- | --- | --- | --- | --- | --- |
|  |  | Scheduled doses | Actual given doses | Scheduled doses | Actual given doses | Scheduled doses | Actual given doses | Scheduled doses | Actual given doses | Scheduled doses | Actual given doses |
| **4.6.1** | OPV |  |  |  |  |  |  |  |  |  |  |
| **4.6.2** | HBV |  |  |  |  |  |  |  |  |  |  |
| **4.6.3** | BCG |  |  |  |  |  |  |  |  |  |  |
| **4.6.4** | Pentavalent |  |  |  |  |  |  |  |  |  |  |
| **4.6.5** | DPT |  |  |  |  |  |  |  |  |  |  |
| **4.6.6** | Rotavirus |  |  |  |  |  |  |  |  |  |  |
| **4.6.7** | IPV |  |  |  |  |  |  |  |  |  |  |
| **4.6.8** | Measles |  |  |  |  |  |  |  |  |  |  |
| **4.6.9** | MR |  |  |  |  |  |  |  |  |  |  |
| **4.6.10** | TT |  |  |  |  |  |  |  |  |  |  |
| **4.6.11** | PCV |  |  |  |  |  |  |  |  |  |  |
| **4.6.12** | JE |  |  |  |  |  |  |  |  |  |  |
| **4.6.13** | MMR |  |  |  |  |  |  |  |  |  |  |

**4.7 Please record the number of beneficiaries received vaccines at the session sites for the month July 2016**

|  |  | **Session 1** | | **Session 2** | | **Session 3** | | **Session 4** | | **Session 5** | |
| --- | --- | --- | --- | --- | --- | --- | --- | --- | --- | --- | --- |
|  |  | Scheduled doses | Actual given doses | Scheduled doses | Actual given doses | Scheduled doses | Actual given doses | Scheduled doses | Actual given doses | Scheduled doses | Actual given doses |
| **4.7.1** | OPV |  |  |  |  |  |  |  |  |  |  |
| **4.7.2** | HBV |  |  |  |  |  |  |  |  |  |  |
| **4.7.3** | BCG |  |  |  |  |  |  |  |  |  |  |
| **4.7.4** | Pentavalent |  |  |  |  |  |  |  |  |  |  |
| **4.7.5** | DPT |  |  |  |  |  |  |  |  |  |  |
| **4.7.6** | Rotavirus |  |  |  |  |  |  |  |  |  |  |
| **4.7.7** | IPV |  |  |  |  |  |  |  |  |  |  |
| **4.7.8** | Measles |  |  |  |  |  |  |  |  |  |  |
| **4.7.9** | MR |  |  |  |  |  |  |  |  |  |  |
| **4.7.10** | TT |  |  |  |  |  |  |  |  |  |  |
| **4.7.11** | PCV |  |  |  |  |  |  |  |  |  |  |
| **4.7.12** | JE |  |  |  |  |  |  |  |  |  |  |
| **4.7.13** | MMR |  |  |  |  |  |  |  |  |  |  |

**4.8 Please record the number of beneficiaries received vaccines at the session sites for the month August 2016**

|  |  | **Session 1** | | **Session 2** | | **Session 3** | | **Session 4** | | **Session 5** | |
| --- | --- | --- | --- | --- | --- | --- | --- | --- | --- | --- | --- |
|  |  | Scheduled doses | Actual given doses | Scheduled doses | Actual given doses | Scheduled doses | Actual given doses | Scheduled doses | Actual given doses | Scheduled doses | Actual given doses |
| **4.8.1** | OPV |  |  |  |  |  |  |  |  |  |  |
| **4.8.2** | HBV |  |  |  |  |  |  |  |  |  |  |
| **4.8.3** | BCG |  |  |  |  |  |  |  |  |  |  |
| **4.8.4** | Pentavalent |  |  |  |  |  |  |  |  |  |  |
| **4.8.5** | DPT |  |  |  |  |  |  |  |  |  |  |
| **4.8.6** | Rotavirus |  |  |  |  |  |  |  |  |  |  |
| **4.8.7** | IPV |  |  |  |  |  |  |  |  |  |  |
| **4.8.8** | Measles |  |  |  |  |  |  |  |  |  |  |
| **4.8.9** | MR |  |  |  |  |  |  |  |  |  |  |
| **4.8.10** | TT |  |  |  |  |  |  |  |  |  |  |
| **4.8.11** | PCV |  |  |  |  |  |  |  |  |  |  |
| **4.8.12** | JE |  |  |  |  |  |  |  |  |  |  |
| **4.8.13** | MMR |  |  |  |  |  |  |  |  |  |  |

**4.9 Please record the number of beneficiaries received vaccines at the session sites for the month September 2016**

|  |  | **Session 1** | | **Session 2** | | **Session 3** | | **Session 4** | | **Session 5** | |
| --- | --- | --- | --- | --- | --- | --- | --- | --- | --- | --- | --- |
|  |  | Scheduled doses | Actual given doses | Scheduled doses | Actual given doses | Scheduled doses | Actual given doses | Scheduled doses | Actual given doses | Scheduled doses | Actual given doses |
| **4.9.1** | OPV |  |  |  |  |  |  |  |  |  |  |
| **4.9.2** | HBV |  |  |  |  |  |  |  |  |  |  |
| **4.9.3** | BCG |  |  |  |  |  |  |  |  |  |  |
| **4.9.4** | Pentavalent |  |  |  |  |  |  |  |  |  |  |
| **4.9.5** | DPT |  |  |  |  |  |  |  |  |  |  |
| **4.9.6** | Rotavirus |  |  |  |  |  |  |  |  |  |  |
| **4.9.7** | IPV |  |  |  |  |  |  |  |  |  |  |
| **4.9.8** | Measles |  |  |  |  |  |  |  |  |  |  |
| **4.9.9** | MR |  |  |  |  |  |  |  |  |  |  |
| **4.9.10** | TT |  |  |  |  |  |  |  |  |  |  |
| **4.9.11** | PCV |  |  |  |  |  |  |  |  |  |  |
| **4.9.12** | JE |  |  |  |  |  |  |  |  |  |  |
| **4.9.13** | MMR |  |  |  |  |  |  |  |  |  |  |

**4.10 Please record the number of beneficiaries received vaccines at the session sites for the month October 2016**

|  |  | **Session 1** | | **Session 2** | | **Session 3** | | **Session 4** | | **Session 5** | |
| --- | --- | --- | --- | --- | --- | --- | --- | --- | --- | --- | --- |
|  |  | Scheduled doses | Actual given doses | Scheduled doses | Actual given doses | Scheduled doses | Actual given doses | Scheduled doses | Actual given doses | Scheduled doses | Actual given doses |
| **4.10.1** | OPV |  |  |  |  |  |  |  |  |  |  |
| **4.10.2** | HBV |  |  |  |  |  |  |  |  |  |  |
| **4.10.3** | BCG |  |  |  |  |  |  |  |  |  |  |
| **4.10.4** | Pentavalent |  |  |  |  |  |  |  |  |  |  |
| **4.10.5** | DPT |  |  |  |  |  |  |  |  |  |  |
| **4.10.6** | Rotavirus |  |  |  |  |  |  |  |  |  |  |
| **4.10.7** | IPV |  |  |  |  |  |  |  |  |  |  |
| **4.10.8** | Measles |  |  |  |  |  |  |  |  |  |  |
| **4.10.9** | MR |  |  |  |  |  |  |  |  |  |  |
| **4.10.10** | TT |  |  |  |  |  |  |  |  |  |  |
| **4.10.11** | PCV |  |  |  |  |  |  |  |  |  |  |
| **4.10.12** | JE |  |  |  |  |  |  |  |  |  |  |
| **4.10.13** | MMR |  |  |  |  |  |  |  |  |  |  |

**4.11 Please record the number of beneficiaries received vaccines at the session sites for the month November 2016**

|  |  | **Session 1** | | **Session 2** | | **Session 3** | | **Session 4** | | **Session 5** | |
| --- | --- | --- | --- | --- | --- | --- | --- | --- | --- | --- | --- |
|  |  | Scheduled doses | Actual given doses | Scheduled doses | Actual given doses | Scheduled doses | Actual given doses | Scheduled doses | Actual given doses | Scheduled doses | Actual given doses |
| **4.11.1** | OPV |  |  |  |  |  |  |  |  |  |  |
| **4.11.2** | HBV |  |  |  |  |  |  |  |  |  |  |
| **4.11.3** | BCG |  |  |  |  |  |  |  |  |  |  |
| **4.11.4** | Pentavalent |  |  |  |  |  |  |  |  |  |  |
| **4.11.5** | DPT |  |  |  |  |  |  |  |  |  |  |
| **4.11.6** | Rotavirus |  |  |  |  |  |  |  |  |  |  |
| **4.11.7** | IPV |  |  |  |  |  |  |  |  |  |  |
| **4.11.8** | Measles |  |  |  |  |  |  |  |  |  |  |
| **4.11.9** | MR |  |  |  |  |  |  |  |  |  |  |
| **4.11.10** | TT |  |  |  |  |  |  |  |  |  |  |
| **4.11.11** | PCV |  |  |  |  |  |  |  |  |  |  |
| **4.11.12** | JE |  |  |  |  |  |  |  |  |  |  |
| **4.11.13** | MMR |  |  |  |  |  |  |  |  |  |  |

**4.12 Please record the number of beneficiaries received vaccines at the session sites for the month December 2016**

|  |  | **Session 1** | | **Session 2** | | **Session 3** | | **Session 4** | | **Session 5** | |
| --- | --- | --- | --- | --- | --- | --- | --- | --- | --- | --- | --- |
|  |  | Scheduled doses | Actual given doses | Scheduled doses | Actual given doses | Scheduled doses | Actual given doses | Scheduled doses | Actual given doses | Scheduled doses | Actual given doses |
| **4.12.1** | OPV |  |  |  |  |  |  |  |  |  |  |
| **4.12.2** | HBV |  |  |  |  |  |  |  |  |  |  |
| **4.12.3** | BCG |  |  |  |  |  |  |  |  |  |  |
| **4.12.4** | Pentavalent |  |  |  |  |  |  |  |  |  |  |
| **4.12.5** | DPT |  |  |  |  |  |  |  |  |  |  |
| **4.12.6** | Rotavirus |  |  |  |  |  |  |  |  |  |  |
| **4.12.7** | IPV |  |  |  |  |  |  |  |  |  |  |
| **4.12.8** | Measles |  |  |  |  |  |  |  |  |  |  |
| **4.12.9** | MR |  |  |  |  |  |  |  |  |  |  |
| **4.12.10** | TT |  |  |  |  |  |  |  |  |  |  |
| **4.12.11** | PCV |  |  |  |  |  |  |  |  |  |  |
| **4.12.12** | JE |  |  |  |  |  |  |  |  |  |  |
| **4.12.13** | MMR |  |  |  |  |  |  |  |  |  |  |

**4.13 Please record the number of beneficiaries received vaccines at the session sites for the month January 2017**

|  |  | **Session 1** | | **Session 2** | | **Session 3** | | **Session 4** | | **Session 5** | |
| --- | --- | --- | --- | --- | --- | --- | --- | --- | --- | --- | --- |
|  |  | Scheduled doses | Actual given doses | Scheduled doses | Actual given doses | Scheduled doses | Actual given doses | Scheduled doses | Actual given doses | Scheduled doses | Actual given doses |
| **4.13.1** | OPV |  |  |  |  |  |  |  |  |  |  |
| **4.13.2** | HBV |  |  |  |  |  |  |  |  |  |  |
| **4.13.3** | BCG |  |  |  |  |  |  |  |  |  |  |
| **4.13.4** | Pentavalent |  |  |  |  |  |  |  |  |  |  |
| **4.13.5** | DPT |  |  |  |  |  |  |  |  |  |  |
| **4.13.6** | Rotavirus |  |  |  |  |  |  |  |  |  |  |
| **4.13.7** | IPV |  |  |  |  |  |  |  |  |  |  |
| **4.13.8** | Measles |  |  |  |  |  |  |  |  |  |  |
| **4.13.9** | MR |  |  |  |  |  |  |  |  |  |  |
| **4.13.10** | TT |  |  |  |  |  |  |  |  |  |  |
| **4.13.11** | PCV |  |  |  |  |  |  |  |  |  |  |
| **4.13.12** | JE |  |  |  |  |  |  |  |  |  |  |
| **4.13.13** | MMR |  |  |  |  |  |  |  |  |  |  |

**4.14 Please record the number of beneficiaries received vaccines at the session sites for the month February 2017**

|  |  | **Session 1** | | **Session 2** | | **Session 3** | | **Session 4** | | **Session 5** | |
| --- | --- | --- | --- | --- | --- | --- | --- | --- | --- | --- | --- |
|  |  | Scheduled doses | Actual given doses | Scheduled doses | Actual given doses | Scheduled doses | Actual given doses | Scheduled doses | Actual given doses | Scheduled doses | Actual given doses |
| **4.14.1** | OPV |  |  |  |  |  |  |  |  |  |  |
| **4.14.2** | HBV |  |  |  |  |  |  |  |  |  |  |
| **4.14.3** | BCG |  |  |  |  |  |  |  |  |  |  |
| **4.14.4** | Pentavalent |  |  |  |  |  |  |  |  |  |  |
| **4.14.5** | DPT |  |  |  |  |  |  |  |  |  |  |
| **4.14.6** | Rotavirus |  |  |  |  |  |  |  |  |  |  |
| **4.14.7** | IPV |  |  |  |  |  |  |  |  |  |  |
| **4.14.8** | Measles |  |  |  |  |  |  |  |  |  |  |
| **4.14.9** | MR |  |  |  |  |  |  |  |  |  |  |
| **4.14.10** | TT |  |  |  |  |  |  |  |  |  |  |
| **4.14.11** | PCV |  |  |  |  |  |  |  |  |  |  |
| **4.14.12** | JE |  |  |  |  |  |  |  |  |  |  |
| **4.14.13** | MMR |  |  |  |  |  |  |  |  |  |  |

**4.15 Please record the number of beneficiaries received vaccines at the session sites for the month March 2017**

|  |  | **Session 1** | | **Session 2** | | **Session 3** | | **Session 4** | | **Session 5** | |
| --- | --- | --- | --- | --- | --- | --- | --- | --- | --- | --- | --- |
|  |  | Scheduled doses | Actual given doses | Scheduled doses | Actual given doses | Scheduled doses | Actual given doses | Scheduled doses | Actual given doses | Scheduled doses | Actual given doses |
| **4.15.1** | OPV |  |  |  |  |  |  |  |  |  |  |
| **4.15.2** | HBV |  |  |  |  |  |  |  |  |  |  |
| **4.15.3** | BCG |  |  |  |  |  |  |  |  |  |  |
| **4.15.4** | Pentavalent |  |  |  |  |  |  |  |  |  |  |
| **4.15.5** | DPT |  |  |  |  |  |  |  |  |  |  |
| **4.15.6** | Rotavirus |  |  |  |  |  |  |  |  |  |  |
| **4.15.7** | IPV |  |  |  |  |  |  |  |  |  |  |
| **4.15.8** | Measles |  |  |  |  |  |  |  |  |  |  |
| **4.15.9** | MR |  |  |  |  |  |  |  |  |  |  |
| **4.15.10** | TT |  |  |  |  |  |  |  |  |  |  |
| **4.15.11** | PCV |  |  |  |  |  |  |  |  |  |  |
| **4.15.12** | JE |  |  |  |  |  |  |  |  |  |  |
| **4.15.13** | MMR |  |  |  |  |  |  |  |  |  |  |

**4.16 Please record the number of beneficiaries received vaccines at the session sites for the month April 2017**

|  |  | **Session 1** | | **Session 2** | | **Session 3** | | **Session 4** | | **Session 5** | |
| --- | --- | --- | --- | --- | --- | --- | --- | --- | --- | --- | --- |
|  |  | Scheduled doses | Actual given doses | Scheduled doses | Actual given doses | Scheduled doses | Actual given doses | Scheduled doses | Actual given doses | Scheduled doses | Actual given doses |
| **4.16.1** | OPV |  |  |  |  |  |  |  |  |  |  |
| **4.16.2** | HBV |  |  |  |  |  |  |  |  |  |  |
| **4.16.3** | BCG |  |  |  |  |  |  |  |  |  |  |
| **4.16.4** | Pentavalent |  |  |  |  |  |  |  |  |  |  |
| **4.16.5** | DPT |  |  |  |  |  |  |  |  |  |  |
| **4.16.6** | Rotavirus |  |  |  |  |  |  |  |  |  |  |
| **4.16.7** | IPV |  |  |  |  |  |  |  |  |  |  |
| **4.16.8** | Measles |  |  |  |  |  |  |  |  |  |  |
| **4.16.9** | MR |  |  |  |  |  |  |  |  |  |  |
| **4.16.10** | TT |  |  |  |  |  |  |  |  |  |  |
| **4.16.11** | PCV |  |  |  |  |  |  |  |  |  |  |
| **4.16.12** | JE |  |  |  |  |  |  |  |  |  |  |
| **4.16.13** | MMR |  |  |  |  |  |  |  |  |  |  |

**4.17 Please record the number of beneficiaries received vaccines at the session sites for the month May 2017**

|  |  | **Session 1** | | **Session 2** | | **Session 3** | | **Session 4** | | **Session 5** | |
| --- | --- | --- | --- | --- | --- | --- | --- | --- | --- | --- | --- |
|  |  | Scheduled doses | Actual given doses | Scheduled doses | Actual given doses | Scheduled doses | Actual given doses | Scheduled doses | Actual given doses | Scheduled doses | Actual given doses |
| **4.17.1** | OPV |  |  |  |  |  |  |  |  |  |  |
| **4.17.2** | HBV |  |  |  |  |  |  |  |  |  |  |
| **4.17.3** | BCG |  |  |  |  |  |  |  |  |  |  |
| **4.17.4** | Pentavalent |  |  |  |  |  |  |  |  |  |  |
| **4.17.5** | DPT |  |  |  |  |  |  |  |  |  |  |
| **4.17.6** | Rotavirus |  |  |  |  |  |  |  |  |  |  |
| **4.17.7** | IPV |  |  |  |  |  |  |  |  |  |  |
| **4.17.8** | Measles |  |  |  |  |  |  |  |  |  |  |
| **4.17.9** | MR |  |  |  |  |  |  |  |  |  |  |
| **4.17.10** | TT |  |  |  |  |  |  |  |  |  |  |
| **4.17.11** | PCV |  |  |  |  |  |  |  |  |  |  |
| **4.17.12** | JE |  |  |  |  |  |  |  |  |  |  |
| **4.17.13** | MMR |  |  |  |  |  |  |  |  |  |  |

**4.18 Please record the number of beneficiaries received vaccines at the session sites for the month June 2017**

|  |  | **Session 1** | | **Session 2** | | **Session 3** | | **Session 4** | | **Session 5** | |
| --- | --- | --- | --- | --- | --- | --- | --- | --- | --- | --- | --- |
|  |  | Scheduled doses | Actual given doses | Scheduled doses | Actual given doses | Scheduled doses | Scheduled doses | Scheduled doses | Actual given doses | Scheduled doses | Actual given doses |
| **4.18.1** | OPV |  |  |  |  |  |  |  |  |  |  |
| **4.18.2** | HBV |  |  |  |  |  |  |  |  |  |  |
| **4.18.3** | BCG |  |  |  |  |  |  |  |  |  |  |
| **4.18.4** | Pentavalent |  |  |  |  |  |  |  |  |  |  |
| **4.18.5** | DPT |  |  |  |  |  |  |  |  |  |  |
| **4.18.6** | Rotavirus |  |  |  |  |  |  |  |  |  |  |
| **4.18.7** | IPV |  |  |  |  |  |  |  |  |  |  |
| **4.18.8** | Measles |  |  |  |  |  |  |  |  |  |  |
| **4.18.9** | MR |  |  |  |  |  |  |  |  |  |  |
| **4.18.10** | TT |  |  |  |  |  |  |  |  |  |  |
| **4.18.11** | PCV |  |  |  |  |  |  |  |  |  |  |
| **4.18.12** | JE |  |  |  |  |  |  |  |  |  |  |
| **4.18.13** | MMR |  |  |  |  |  |  |  |  |  |  |

**4.19 Please record the number of beneficiaries received vaccines at the session sites for the month July 2017**

|  |  | **Session 1** | | **Session 2** | | **Session 3** | | **Session 4** | | **Session 4** | |
| --- | --- | --- | --- | --- | --- | --- | --- | --- | --- | --- | --- |
|  |  | Scheduled doses | Actual given doses | Scheduled doses | Actual given doses | Scheduled doses | Actual given doses | Scheduled doses | Actual given doses | Scheduled doses | Actual given doses |
| **4.19.1** | OPV |  |  |  |  |  |  |  |  |  |  |
| **4.19.2** | HBV |  |  |  |  |  |  |  |  |  |  |
| **4.19.3** | BCG |  |  |  |  |  |  |  |  |  |  |
| **4.19.4** | Pentavalent |  |  |  |  |  |  |  |  |  |  |
| **4.19.5** | DPT |  |  |  |  |  |  |  |  |  |  |
| **4.19.6** | Rotavirus |  |  |  |  |  |  |  |  |  |  |
| **4.19.7** | IPV |  |  |  |  |  |  |  |  |  |  |
| **4.19.8** | Measles |  |  |  |  |  |  |  |  |  |  |
| **4.19.9** | MR |  |  |  |  |  |  |  |  |  |  |
| **4.19.10** | TT |  |  |  |  |  |  |  |  |  |  |
| **4.19.11** | PCV |  |  |  |  |  |  |  |  |  |  |
| **4.19.12** | JE |  |  |  |  |  |  |  |  |  |  |
| **4.19.13** | MMR |  |  |  |  |  |  |  |  |  |  |

**4.20 Please record the number of beneficiaries received vaccines at the session sites for the month August 2017**

|  |  | **Session 1** | | **Session 2** | | **Session 3** | | **Session 4** | | **Session 5** | |
| --- | --- | --- | --- | --- | --- | --- | --- | --- | --- | --- | --- |
|  |  | Scheduled doses | Actual given doses | Scheduled doses | Actual given doses | Scheduled doses | Actual given doses | Scheduled doses | Actual given doses | Scheduled doses | Actual given doses |
| **4.20.1** | OPV |  |  |  |  |  |  |  |  |  |  |
| **4.20.2** | HBV |  |  |  |  |  |  |  |  |  |  |
| **4.20.3** | BCG |  |  |  |  |  |  |  |  |  |  |
| **4.20.4** | Pentavalent |  |  |  |  |  |  |  |  |  |  |
| **4.20.5** | DPT |  |  |  |  |  |  |  |  |  |  |
| **4.20.6** | Rotavirus |  |  |  |  |  |  |  |  |  |  |
| **4.20.7** | IPV |  |  |  |  |  |  |  |  |  |  |
| **4.20.8** | Measles |  |  |  |  |  |  |  |  |  |  |
| **4.20.9** | MR |  |  |  |  |  |  |  |  |  |  |
| **4.20.10** | TT |  |  |  |  |  |  |  |  |  |  |
| **4.20.11** | PCV |  |  |  |  |  |  |  |  |  |  |
| **4.20.12** | JE |  |  |  |  |  |  |  |  |  |  |
| **4.20.13** | MMR |  |  |  |  |  |  |  |  |  |  |

**4.21 Please record the number of beneficiaries received vaccines at the session sites for the month September 2017**

|  |  | **Session 1** | | **Session 2** | | **Session 3** | | **Session 4** | | **Session 5** | |
| --- | --- | --- | --- | --- | --- | --- | --- | --- | --- | --- | --- |
|  |  | Scheduled doses | Actual given doses | Scheduled doses | Actual given doses | Scheduled doses | Actual given doses | Scheduled doses | Actual given doses | Scheduled doses | Actual given doses |
| **4.21.1** | OPV |  |  |  |  |  |  |  |  |  |  |
| **4.21.2** | HBV |  |  |  |  |  |  |  |  |  |  |
| **4.21.3** | BCG |  |  |  |  |  |  |  |  |  |  |
| **4.21.4** | Pentavalent |  |  |  |  |  |  |  |  |  |  |
| **4.21.5** | DPT |  |  |  |  |  |  |  |  |  |  |
| **4.21.6** | Rotavirus |  |  |  |  |  |  |  |  |  |  |
| **4.21.7** | IPV |  |  |  |  |  |  |  |  |  |  |
| **4.21.8** | Measles |  |  |  |  |  |  |  |  |  |  |
| **4.21.9** | MR |  |  |  |  |  |  |  |  |  |  |
| **4.21.10** | TT |  |  |  |  |  |  |  |  |  |  |
| **4.21.11** | PCV |  |  |  |  |  |  |  |  |  |  |
| **4.21.12** | JE |  |  |  |  |  |  |  |  |  |  |
| **4.21.13** | MMR |  |  |  |  |  |  |  |  |  |  |

**4.22 Please record the number of beneficiaries received vaccines at the session sites for the month October 2017**

|  |  | **Session 1** | | **Session 2** | | **Session 3** | | **Session 4** | | **Session 5** | |
| --- | --- | --- | --- | --- | --- | --- | --- | --- | --- | --- | --- |
|  |  | Scheduled doses | Actual given doses | Scheduled doses | Actual given doses | Scheduled doses | Actual given doses | Scheduled doses | Actual given doses | Scheduled doses | Actual given doses |
| **4.22.1** | OPV |  |  |  |  |  |  |  |  |  |  |
| **4.22.2** | HBV |  |  |  |  |  |  |  |  |  |  |
| **4.22.3** | BCG |  |  |  |  |  |  |  |  |  |  |
| **4.22.4** | Pentavalent |  |  |  |  |  |  |  |  |  |  |
| **4.22.5** | DPT |  |  |  |  |  |  |  |  |  |  |
| **4.22.6** | Rotavirus |  |  |  |  |  |  |  |  |  |  |
| **4.22.7** | IPV |  |  |  |  |  |  |  |  |  |  |
| **4.22.8** | Measles |  |  |  |  |  |  |  |  |  |  |
| **4.22.9** | MR |  |  |  |  |  |  |  |  |  |  |
| **4.22.10** | TT |  |  |  |  |  |  |  |  |  |  |
| **4.22.11** | PCV |  |  |  |  |  |  |  |  |  |  |
| **4.22.12** | JE |  |  |  |  |  |  |  |  |  |  |
| **4.22.13** | MMR |  |  |  |  |  |  |  |  |  |  |

**4.23 Please record the number of beneficiaries received vaccines at the session sites for the month November 2017**

|  |  | **Session 1** | | **Session 2** | | **Session 3** | | **Session 4** | | **Session 5** | |
| --- | --- | --- | --- | --- | --- | --- | --- | --- | --- | --- | --- |
|  |  | Scheduled doses | Actual given doses | Scheduled doses | Actual given doses | Scheduled doses | Actual given doses | Scheduled doses | Actual given doses | Scheduled doses | Actual given doses |
| **4.23.1** | OPV |  |  |  |  |  |  |  |  |  |  |
| **4.23.2** | HBV |  |  |  |  |  |  |  |  |  |  |
| **4.23.3** | BCG |  |  |  |  |  |  |  |  |  |  |
| **4.23.4** | Pentavalent |  |  |  |  |  |  |  |  |  |  |
| **4.23.5** | DPT |  |  |  |  |  |  |  |  |  |  |
| **4.23.6** | Rotavirus |  |  |  |  |  |  |  |  |  |  |
| **4.23.7** | IPV |  |  |  |  |  |  |  |  |  |  |
| **4.23.8** | Measles |  |  |  |  |  |  |  |  |  |  |
| **4.23.9** | MR |  |  |  |  |  |  |  |  |  |  |
| **4.23.10** | TT |  |  |  |  |  |  |  |  |  |  |
| **4.23.11** | PCV |  |  |  |  |  |  |  |  |  |  |
| **4.23.12** | JE |  |  |  |  |  |  |  |  |  |  |
| **4.23.13** | MMR |  |  |  |  |  |  |  |  |  |  |

**4.24 Please record the number of beneficiaries received vaccines at the session sites for the month December 2017**

|  |  | **Session 1** | | **Session 2** | | **Session 3** | | **Session 4** | | **Session 5** | |
| --- | --- | --- | --- | --- | --- | --- | --- | --- | --- | --- | --- |
|  |  | Scheduled doses | Actual given doses | Scheduled doses | Actual given doses | Scheduled doses | Actual given doses | Scheduled doses | Actual given doses | Scheduled doses | Actual given doses |
| **4.24.1** | OPV |  |  |  |  |  |  |  |  |  |  |
| **4.24.2** | HBV |  |  |  |  |  |  |  |  |  |  |
| **4.24.3** | BCG |  |  |  |  |  |  |  |  |  |  |
| **4.24.4** | Pentavalent |  |  |  |  |  |  |  |  |  |  |
| **4.24.5** | DPT |  |  |  |  |  |  |  |  |  |  |
| **4.24.6** | Rotavirus |  |  |  |  |  |  |  |  |  |  |
| **4.24.7** | IPV |  |  |  |  |  |  |  |  |  |  |
| **4.24.8** | Measles |  |  |  |  |  |  |  |  |  |  |
| **4.24.9** | MR |  |  |  |  |  |  |  |  |  |  |
| **4.24.10** | TT |  |  |  |  |  |  |  |  |  |  |
| **4.24.11** | PCV |  |  |  |  |  |  |  |  |  |  |
| **4.24.12** | JE |  |  |  |  |  |  |  |  |  |  |
| **4.24.13** | MMR |  |  |  |  |  |  |  |  |  |  |

**Section D: Monthly report on vaccination from the sub-center**

**Q 5. Please record the vaccination report from the sub-center for each month during January 2016 to December 2017**

| Q no | Beneficiary | Jan 2016 | Feb 2016 | Mar 2016 | Apr 2016 | May 2016 | Jun 2016 | Jul 2016 | Aug 2016 | Sep 2016 |
| --- | --- | --- | --- | --- | --- | --- | --- | --- | --- | --- |
| 5.1 | TT (PW: 1+2+B); Child: 1+2) |  |  |  |  |  |  |  |  |  |
| 5.2 | BCG |  |  |  |  |  |  |  |  |  |
| 5.3 | DPT (1 +2+3) |  |  |  |  |  |  |  |  |  |
| 5.4 | Pentavalenet (1+2+3) |  |  |  |  |  |  |  |  |  |
| 5.5 | OPV (Birth+1+2+3) |  |  |  |  |  |  |  |  |  |
| 5.6 | Hepatitis B (0+1+2+3) |  |  |  |  |  |  |  |  |  |
| 5.7 | IPV (1+2) |  |  |  |  |  |  |  |  |  |
| 5.8 | Rotavirus (1+2+3) |  |  |  |  |  |  |  |  |  |
| 5.9 | Measles (1+Booster) |  |  |  |  |  |  |  |  |  |
| 5.10 | MR (1+Booster) |  |  |  |  |  |  |  |  |  |
| 5.11 | JE (1 +Booster) |  |  |  |  |  |  |  |  |  |
| 5.12 | MMR (1+2) |  |  |  |  |  |  |  |  |  |
| 5.13 | PCV (1+2+3) |  |  |  |  |  |  |  |  |  |
| Q no | Beneficiary | Oct 2016 | Nov 2016 | Dec 2016 | Jan 2017 | Feb 2017 | Mar 2017 | Apr 2017 | May 2017 | Jun 2017 |
| 5.1 | TT (PW: 1+2+ B); Child: 1+2) |  |  |  |  |  |  |  |  |  |
| 5.2 | BCG |  |  |  |  |  |  |  |  |  |
| 5.3 | DPT (1 +2+3) |  |  |  |  |  |  |  |  |  |
| 5.4 | Pentavalenet (1+2+3) |  |  |  |  |  |  |  |  |  |
| 5.5 | OPV (Birth+1+2+3) |  |  |  |  |  |  |  |  |  |
| 5.6 | Hepatitis B (0+1+2+3) |  |  |  |  |  |  |  |  |  |
| 5.7 | IPV (1+2) |  |  |  |  |  |  |  |  |  |
| 5.8 | Rotavirus (1+2+3) |  |  |  |  |  |  |  |  |  |
| 5.9 | Measles (1+Booster) |  |  |  |  |  |  |  |  |  |
| 5.10 | MR (1+Booster) |  |  |  |  |  |  |  |  |  |
| 5.11 | JE (1 +Booster) |  |  |  |  |  |  |  |  |  |
| 5.12 | MMR (1+2) |  |  |  |  |  |  |  |  |  |
| 5.13 | PCV (1+2+3) |  |  |  |  |  |  |  |  |  |

**Please record the vaccination report from the sub-center for each month during January 2016 to December 2017**

| Q no | Beneficiary | Jul 2017 | Aug 2017 | Sep 2017 | Oct 2017 | Nov 2017 | Dec 2017 |  |  |  |
| --- | --- | --- | --- | --- | --- | --- | --- | --- | --- | --- |
| 5.1 | TT (PW:1+2+B); Child: 1+2) |  |  |  |  |  |  |  |  |  |
| 5.2 | BCG |  |  |  |  |  |  |  |  |  |
| 5.3 | DPT (1 +2+3) |  |  |  |  |  |  |  |  |  |
| 5.4 | Pentavalenet (1+2+3) |  |  |  |  |  |  |  |  |  |
| 5.5 | OPV (Birth+1+2+3) |  |  |  |  |  |  |  |  |  |
| 5.6 | Hepatitis B (0+1+2+3) |  |  |  |  |  |  |  |  |  |
| 5.7 | IPV (1+2) |  |  |  |  |  |  |  |  |  |
| 5.8 | Rotavirus (1+2+3) |  |  |  |  |  |  |  |  |  |
| 5.9 | Measles (1+Booster) |  |  |  |  |  |  |  |  |  |
| 5.10 | MR (1+Booster) |  |  |  |  |  |  |  |  |  |
| 5.11 | JE (1 +Booster) |  |  |  |  |  |  |  |  |  |
| 5.12 | MMR (1+2) |  |  |  |  |  |  |  |  |  |
| 5.13 | PCV (1+2+3) |  |  |  |  |  |  |  |  |  |

**Any specific comments/ observations/feedback.**

**Thank the ANM/ vaccinator for her/his support.**
