# Supplementary material for: Documentation of vaccine wastage in two different geographic contexts under the universal immunization program in India
Source: BMC Public Health. 2020 Apr 25;20:556. doi: 10.1186/s12889-020-08637-1 (PMC7183620; doi:10.1186/s12889-020-08637-1)
Supplement: Supplementary file 2 — Additional file 2: Supplementary file 2. Vaccine wastage documentation tool at facility level [file 12889_2020_8637_MOESM2_ESM.docx]

| **UNIQUE ID** |  |
| --- | --- |

**Documentation of vaccine wastage under the universal immunization program in the districts of Kangra (Himachal Pradesh) and Pune (Maharashtra)**

**Vaccine Utilization Documentation Tool (VUDT)**

**(For Vaccine Stores)**

| - 1. **District Name** | **Kangra Pune** |
| --- | --- |
| - 1. **Block Name** |  |
| - 1. **Name of the Facility** |  |
| **1.4 Type/Level of Vaccine Store**  1- District, 2- SDH/RH/ZH; 3-CHC;  4- PHC/UPHC; 9- Other | If other, specify ………………………………… |
| **1.5 Date of assessment/visit** |  |

| **1.6** | **Persons/ staff met during the visit**  (write only designations, not names) | 1. |
| --- | --- | --- |
|  |  | 2. |
|  |  | 3. |
|  |  | 4. |
| **1.7** | **Documents reviewed**  (source documents for data collection)  (mark ‘X’ for the reviewed documents) | 1. Vaccine Stock Register |
|  |  | 2. Vaccine Distribution Register |
|  |  | 3. MCH/RCH register |
|  |  | 4. Monthly reports |
|  |  | 5. Other …………………………. |

**Section A: Cold chain capacity and population information**

**Q1. Please record the cold chain devices/equipment at the facility used for vaccine storage.**

|  | **Question** | **Total (n)** | **Functional (n)** | **Non-functional (n)** |
| --- | --- | --- | --- | --- |
| 1.1 | Number of large ILRs |  |  |  |
| 1.2 | Number of small ILRs |  |  |  |
| 1.3 | Number of large DFs |  |  |  |
| 1.4 | Number of small DFs |  |  |  |
| 1.5 | Domestic refrigerators |  |  |  |
| 1.6 | Cold boxes |  |  |  |

*(Record the cold boxes and domestic refrigerators as zero ‘0’ if not used for vaccine storage)*

**Q2. Please record the target population served by this facility.**

|  | **Question** | **Total (n)** | **Year of estimation** |
| --- | --- | --- | --- |
| 2.1 | Total population served |  |  |
| 2.2 | Annual birth cohort |  |  |
| 2.3 | Total pregnant women |  |  |

**Q3. Please record the month-wise vaccine stock status and utilization for all vaccines**

3.1 Month: **January 2016**

| Q no | Vaccine | Start balance  (A) | No of doses received  (B) | No of doses discarded (unopened) (C) | No of doses issued (D) | No of doses returned (E) | No of doses vaccinated (children/  pregnant women) (F) | End balance  G= (A+B)-(C+D-E) |
| --- | --- | --- | --- | --- | --- | --- | --- | --- |
| 3.1.1 | OPV |  |  |  |  |  |  |  |
| 3.1.2 | HBV |  |  |  |  |  |  |  |
| 3.1.3 | BCG |  |  |  |  |  |  |  |
| 3.1.4 | Penta |  |  |  |  |  |  |  |
| 3.1.5 | DPT |  |  |  |  |  |  |  |
| 3.1.6 | Rota |  |  |  |  |  |  |  |
| 3.1.7 | IPV |  |  |  |  |  |  |  |
| 3.1.8 | Measles |  |  |  |  |  |  |  |
| 3.1.9 | MR |  |  |  |  |  |  |  |
| 3.1.10 | TT |  |  |  |  |  |  |  |
| 3.1.11 | PCV |  |  |  |  |  |  |  |
| 3.1.12 | JE |  |  |  |  |  |  |  |
| 3.1.13 | MMR |  |  |  |  |  |  |  |

**Q3. Please record the month-wise vaccine stock status and utilization for all vaccines**

3.2 Month: **February 2016**

| Q no | Vaccine | Start balance  (A) | No of doses received  (B) | No of doses discarded (unopened) (C) | No of doses issued (D) | No of doses returned (E) | No of doses vaccinated (children/  pregnant women) (F) | End balance  G= (A+B)-(C+D-E) |
| --- | --- | --- | --- | --- | --- | --- | --- | --- |
| 3.2.1 | BCG |  |  |  |  |  |  |  |
| 3.2.2 | OPV |  |  |  |  |  |  |  |
| 3.2.3 | HBV |  |  |  |  |  |  |  |
| 3.2.4 | Penta |  |  |  |  |  |  |  |
| 3.2.5 | Rotavirus |  |  |  |  |  |  |  |
| 3.2.6 | DPT |  |  |  |  |  |  |  |
| 3.2.7 | Measles |  |  |  |  |  |  |  |
| 3.2.8 | MR |  |  |  |  |  |  |  |
| 3.2.9 | TT |  |  |  |  |  |  |  |
| 3.2.10 | IPV |  |  |  |  |  |  |  |
| 3.2.11 | PCV |  |  |  |  |  |  |  |
| 3.2.12 | JE |  |  |  |  |  |  |  |
| 3.2.13 | MMR |  |  |  |  |  |  |  |

3.3 Month: **March 2016**

| Q no | Vaccine | Start balance  (A) | No of doses received  (B) | No of doses discarded (unopened) (C) | No of doses issued (D) | No of doses returned (E) | No of doses vaccinated (children/  pregnant women) (F) | End balance  G= (A+B)-(C+D-E) |
| --- | --- | --- | --- | --- | --- | --- | --- | --- |
| 3.3.1 | BCG |  |  |  |  |  |  |  |
| 3.3.2 | OPV |  |  |  |  |  |  |  |
| 3.3.3 | HBV |  |  |  |  |  |  |  |
| 3.3.4 | Penta |  |  |  |  |  |  |  |
| 3.3.5 | Rotavirus |  |  |  |  |  |  |  |
| 3.3.6 | DPT |  |  |  |  |  |  |  |
| 3.3.7 | Measles |  |  |  |  |  |  |  |
| 3.3.8 | MR |  |  |  |  |  |  |  |
| 3.3.9 | TT |  |  |  |  |  |  |  |
| 3.3.10 | IPV |  |  |  |  |  |  |  |
| 3.3.11 | PCV |  |  |  |  |  |  |  |
| 3.3.12 | JE |  |  |  |  |  |  |  |
| 3.3.13 | MMR |  |  |  |  |  |  |  |

3.4 Month: **April** **2016**

| Q no | Vaccine | Start balance  (A) | No of doses received  (B) | No of doses discarded (unopened) (C) | No of doses issued (D) | No of doses returned (E) | No of doses vaccinated (children/  pregnant women) (F) | End balance  G= (A+B)-(C+D-E) |
| --- | --- | --- | --- | --- | --- | --- | --- | --- |
| 3.4.1 | BCG |  |  |  |  |  |  |  |
| 3.4.2 | OPV |  |  |  |  |  |  |  |
| 3.4.3 | HBV |  |  |  |  |  |  |  |
| 3.4.4 | Penta |  |  |  |  |  |  |  |
| 3.4.5 | Rotavirus |  |  |  |  |  |  |  |
| 3.4.6 | DPT |  |  |  |  |  |  |  |
| 3.4.7 | Measles |  |  |  |  |  |  |  |
| 3.4.8 | MR |  |  |  |  |  |  |  |
| 3.4.9 | TT |  |  |  |  |  |  |  |
| 3.4.10 | IPV |  |  |  |  |  |  |  |
| 3.4.11 | PCV |  |  |  |  |  |  |  |
| 3.4.12 | JE |  |  |  |  |  |  |  |
| 3.4.13 | MMR |  |  |  |  |  |  |  |

3.5 Month: **May 2016**

| Q no | Vaccine | Start balance  (A) | No of doses received  (B) | No of doses discarded (unopened) (C) | No of doses issued (D) | No of doses returned (E) | No of doses vaccinated (children/  pregnant women) (F) | End balance  G= (A+B)-(C+D-E) |
| --- | --- | --- | --- | --- | --- | --- | --- | --- |
| 3.5.1 | BCG |  |  |  |  |  |  |  |
| 3.5.2 | OPV |  |  |  |  |  |  |  |
| 3.5.3 | HBV |  |  |  |  |  |  |  |
| 3.5.4 | Penta |  |  |  |  |  |  |  |
| 3.5.5 | Rotavirus |  |  |  |  |  |  |  |
| 3.5.6 | DPT |  |  |  |  |  |  |  |
| 3.5.7 | Measles |  |  |  |  |  |  |  |
| 3.5.8 | MR |  |  |  |  |  |  |  |
| 3.5.9 | TT |  |  |  |  |  |  |  |
| 3.5.10 | IPV |  |  |  |  |  |  |  |
| 3.5.11 | PCV |  |  |  |  |  |  |  |
| 3.5.12 | JE |  |  |  |  |  |  |  |
| 3.5.13 | MMR |  |  |  |  |  |  |  |

3.6 Month: **Jun 2016**

| Q no | Vaccine | Start balance  (A) | No of doses received  (B) | No of doses discarded (unopened) (C) | No of doses issued (D) | No of doses returned (E) | No of doses vaccinated (children/  pregnant women) (F) | End balance  G= (A+B)-(C+D-E) |
| --- | --- | --- | --- | --- | --- | --- | --- | --- |
| 3.6.1 | BCG |  |  |  |  |  |  |  |
| 3.6.2 | OPV |  |  |  |  |  |  |  |
| 3.6.3 | HBV |  |  |  |  |  |  |  |
| 3.6.4 | Penta |  |  |  |  |  |  |  |
| 3.6.5 | Rotavirus |  |  |  |  |  |  |  |
| 3.6.6 | DPT |  |  |  |  |  |  |  |
| 3.6.7 | Measles |  |  |  |  |  |  |  |
| 3.6.8 | MR |  |  |  |  |  |  |  |
| 3.6.9 | TT |  |  |  |  |  |  |  |
| 3.6.10 | IPV |  |  |  |  |  |  |  |
| 3.6.11 | PCV |  |  |  |  |  |  |  |
| 3.6.12 | JE |  |  |  |  |  |  |  |
| 3.6.13 | MMR |  |  |  |  |  |  |  |

3.7 Month: **July** **2016**

| Q no | Vaccine | Start balance  (A) | No of doses received  (B) | No of doses discarded (unopened) (C) | No of doses issued (D) | No of doses returned (E) | No of doses vaccinated (children/  pregnant women) (F) | End balance  G= (A+B)-(C+D-E) |
| --- | --- | --- | --- | --- | --- | --- | --- | --- |
| 3.7.1 | BCG |  |  |  |  |  |  |  |
| 3.7.2 | OPV |  |  |  |  |  |  |  |
| 3.7.3 | HBV |  |  |  |  |  |  |  |
| 3.7.4 | Penta |  |  |  |  |  |  |  |
| 3.7.5 | Rotavirus |  |  |  |  |  |  |  |
| 3.7.6 | DPT |  |  |  |  |  |  |  |
| 3.7.7 | Measles |  |  |  |  |  |  |  |
| 3.7.8 | MR |  |  |  |  |  |  |  |
| 3.7.9 | TT |  |  |  |  |  |  |  |
| 3.7.10 | IPV |  |  |  |  |  |  |  |
| 3.7.11 | PCV |  |  |  |  |  |  |  |
| 3.7.12 | JE |  |  |  |  |  |  |  |
| 3.7.13 | MMR |  |  |  |  |  |  |  |

3.8 Month: **August** **2016**

| Q no | Vaccine | Start balance  (A) | No of doses received  (B) | No of doses discarded (unopened) (C) | No of doses issued (D) | No of doses returned (E) | No of doses vaccinated (children/  pregnant women) (F) | End balance  G= (A+B)-(C+D-E) |
| --- | --- | --- | --- | --- | --- | --- | --- | --- |
| 3.8.1 | BCG |  |  |  |  |  |  |  |
| 3.8.2 | OPV |  |  |  |  |  |  |  |
| 3.8.3 | HBV |  |  |  |  |  |  |  |
| 3.8.4 | Penta |  |  |  |  |  |  |  |
| 3.8.5 | Rotavirus |  |  |  |  |  |  |  |
| 3.8.6 | DPT |  |  |  |  |  |  |  |
| 3.8.7 | Measles |  |  |  |  |  |  |  |
| 3.8.8 | MR |  |  |  |  |  |  |  |
| 3.8.9 | TT |  |  |  |  |  |  |  |
| 3.8.10 | IPV |  |  |  |  |  |  |  |
| 3.8.11 | PCV |  |  |  |  |  |  |  |
| 3.8.12 | JE |  |  |  |  |  |  |  |
| 3.8.13 | MMR |  |  |  |  |  |  |  |

3.9 Month: **September** **2016**

| Q no | Vaccine | Start balance  (A) | No of doses received  (B) | No of doses discarded (unopened) (C) | No of doses issued (D) | No of doses returned (E) | No of doses vaccinated (children/  pregnant women) (F) | End balance  G= (A+B)-(C+D-E) |
| --- | --- | --- | --- | --- | --- | --- | --- | --- |
| 3.9.1 | BCG |  |  |  |  |  |  |  |
| 3.9.2 | OPV |  |  |  |  |  |  |  |
| 3.9.3 | HBV |  |  |  |  |  |  |  |
| 3.9.4 | Penta |  |  |  |  |  |  |  |
| 3.9.5 | Rotavirus |  |  |  |  |  |  |  |
| 3.9.6 | DPT |  |  |  |  |  |  |  |
| 3.9.7 | Measles |  |  |  |  |  |  |  |
| 3.9.8 | MR |  |  |  |  |  |  |  |
| 3.9.9 | TT |  |  |  |  |  |  |  |
| 3.9.10 | IPV |  |  |  |  |  |  |  |
| 3.9.11 | PCV |  |  |  |  |  |  |  |
| 3.9.12 | JE |  |  |  |  |  |  |  |
| 3.9.13 | MMR |  |  |  |  |  |  |  |

3.10 Month: **October** **2016**

| Q no | Vaccine | Start balance  (A) | No of doses received  (B) | No of doses discarded (unopened) (C) | No of doses issued (D) | No of doses returned (E) | No of doses vaccinated (children/  pregnant women) (F) | End balance  G= (A+B)-(C+D-E) |
| --- | --- | --- | --- | --- | --- | --- | --- | --- |
| 3.10.1 | BCG |  |  |  |  |  |  |  |
| 3. 10.2 | OPV |  |  |  |  |  |  |  |
| 3.10.3 | HBV |  |  |  |  |  |  |  |
| 3.10.4 | Penta |  |  |  |  |  |  |  |
| 3.10.5 | Rotavirus |  |  |  |  |  |  |  |
| 3.10.6 | DPT |  |  |  |  |  |  |  |
| 3.10.7 | Measles |  |  |  |  |  |  |  |
| 3.10.8 | MR |  |  |  |  |  |  |  |
| 3.10.9 | TT |  |  |  |  |  |  |  |
| 3.10.10 | IPV |  |  |  |  |  |  |  |
| 3.10.11 | PCV |  |  |  |  |  |  |  |
| 3.10.12 | JE |  |  |  |  |  |  |  |
| 3.10.13 | MMR |  |  |  |  |  |  |  |

3.11 Month: **November** **2016**

| Q no | Vaccine | Start balance  (A) | No of doses received  (B) | No of doses discarded (unopened) (C) | No of doses issued (D) | No of doses returned (E) | No of doses vaccinated (children/  pregnant women) (F) | End balance  G= (A+B)-(C+D-E) |
| --- | --- | --- | --- | --- | --- | --- | --- | --- |
| 3.11.1 | BCG |  |  |  |  |  |  |  |
| 3.11.2 | OPV |  |  |  |  |  |  |  |
| 3.11.3 | HBV |  |  |  |  |  |  |  |
| 3.11.4 | Penta |  |  |  |  |  |  |  |
| 3.11.5 | Rotavirus |  |  |  |  |  |  |  |
| 3.11.6 | DPT |  |  |  |  |  |  |  |
| 3.11.7 | Measles |  |  |  |  |  |  |  |
| 3.11.8 | MR |  |  |  |  |  |  |  |
| 3.11.9 | TT |  |  |  |  |  |  |  |
| 3.11.10 | IPV |  |  |  |  |  |  |  |
| 3.11.11 | PCV |  |  |  |  |  |  |  |
| 3.11.12 | JE |  |  |  |  |  |  |  |
| 3.11.13 | MMR |  |  |  |  |  |  |  |

3.10 Month: **December** **2016**

| Q no | Vaccine | Start balance  (A) | No of doses received  (B) | No of doses discarded (unopened) (C) | No of doses issued (D) | No of doses returned (E) | No of doses vaccinated (children/  pregnant women) (F) | End balance  G= (A+B)-(C+D-E) |
| --- | --- | --- | --- | --- | --- | --- | --- | --- |
| 3.12.1 | BCG |  |  |  |  |  |  |  |
| 3.12.2 | OPV |  |  |  |  |  |  |  |
| 3.12.3 | HBV |  |  |  |  |  |  |  |
| 3.12.4 | Penta |  |  |  |  |  |  |  |
| 3.12.5 | Rotavirus |  |  |  |  |  |  |  |
| 3.12.6 | DPT |  |  |  |  |  |  |  |
| 3.12.7 | Measles |  |  |  |  |  |  |  |
| 3.12.8 | MR |  |  |  |  |  |  |  |
| 3.12.9 | TT |  |  |  |  |  |  |  |
| 3.12.10 | IPV |  |  |  |  |  |  |  |
| 3.12.11 | PCV |  |  |  |  |  |  |  |
| 3.12.12 | JE |  |  |  |  |  |  |  |
| 3.12.13 | MMR |  |  |  |  |  |  |  |

3.13 Month: **January 2017**

| Q no | Vaccine | Start balance  (A) | No of doses received  (B) | No of doses discarded (unopened) (C) | No of doses issued (D) | No of doses returned (E) | No of doses vaccinated (children/  pregnant women) (F) | End balance  G= (A+B)-(C+D-E) |
| --- | --- | --- | --- | --- | --- | --- | --- | --- |
| 3.13.1 | BCG |  |  |  |  |  |  |  |
| 3.13.2 | OPV |  |  |  |  |  |  |  |
| 3.13.3 | HBV |  |  |  |  |  |  |  |
| 3.13.4 | Penta |  |  |  |  |  |  |  |
| 3.13.5 | Rotavirus |  |  |  |  |  |  |  |
| 3.13.6 | DPT |  |  |  |  |  |  |  |
| 3.13.7 | Measles |  |  |  |  |  |  |  |
| 3.13.8 | MR |  |  |  |  |  |  |  |
| 3.13.9 | TT |  |  |  |  |  |  |  |
| 3.13.10 | IPV |  |  |  |  |  |  |  |
| 3.13.11 | PCV |  |  |  |  |  |  |  |
| 3.13.12 | JE |  |  |  |  |  |  |  |
| 3.13.13 | MMR |  |  |  |  |  |  |  |

3.14 Month: **February** **2017**

| Q no | Vaccine | Start balance  (A) | No of doses received  (B) | No of doses discarded (unopened) (C) | No of doses issued (D) | No of doses returned (E) | No of doses vaccinated (children/  pregnant women) (F) | End balance  G= (A+B)-(C+D-E) |
| --- | --- | --- | --- | --- | --- | --- | --- | --- |
| 3.14.1 | BCG |  |  |  |  |  |  |  |
| 3.14.2 | OPV |  |  |  |  |  |  |  |
| 3.14.3 | HBV |  |  |  |  |  |  |  |
| 3.14.4 | Penta |  |  |  |  |  |  |  |
| 3.14.5 | Rotavirus |  |  |  |  |  |  |  |
| 3.14.6 | DPT |  |  |  |  |  |  |  |
| 3.14.7 | Measles |  |  |  |  |  |  |  |
| 3.14.8 | MR |  |  |  |  |  |  |  |
| 3.14.9 | TT |  |  |  |  |  |  |  |
| 3.14.10 | IPV |  |  |  |  |  |  |  |
| 3.14.11 | PCV |  |  |  |  |  |  |  |
| 3.14.12 | JE |  |  |  |  |  |  |  |
| 3.14.13 | MMR |  |  |  |  |  |  |  |

3.15 Month: **March** **2017**

| Q no | Vaccine | Start balance  (A) | No of doses received  (B) | No of doses discarded (unopened) (C) | No of doses issued (D) | No of doses returned (E) | No of doses vaccinated (children/  pregnant women) (F) | End balance  G= (A+B)-(C+D-E) |
| --- | --- | --- | --- | --- | --- | --- | --- | --- |
| 3.15.1 | BCG |  |  |  |  |  |  |  |
| 3.15.2 | OPV |  |  |  |  |  |  |  |
| 3.15.3 | HBV |  |  |  |  |  |  |  |
| 3.15.4 | Penta |  |  |  |  |  |  |  |
| 3.15.5 | Rotavirus |  |  |  |  |  |  |  |
| 3.15.6 | DPT |  |  |  |  |  |  |  |
| 3.15.7 | Measles |  |  |  |  |  |  |  |
| 3.15.8 | MR |  |  |  |  |  |  |  |
| 3.15.9 | TT |  |  |  |  |  |  |  |
| 3.15.10 | IPV |  |  |  |  |  |  |  |
| 3.15.11 | PCV |  |  |  |  |  |  |  |
| 3.15.12 | JE |  |  |  |  |  |  |  |
| 3.15.13 | MMR |  |  |  |  |  |  |  |

3.16 Month: **April** **2017**

| Q no | Vaccine | Start balance  (A) | No of doses received  (B) | No of doses discarded (unopened) (C) | No of doses issued (D) | No of doses returned (E) | No of doses vaccinated (children/  pregnant women) (F) | End balance  G= (A+B)-(C+D-E) |
| --- | --- | --- | --- | --- | --- | --- | --- | --- |
| 3.16.1 | BCG |  |  |  |  |  |  |  |
| 3.16.2 | OPV |  |  |  |  |  |  |  |
| 3.16.3 | HBV |  |  |  |  |  |  |  |
| 3.16.4 | Penta |  |  |  |  |  |  |  |
| 3.16.5 | Rotavirus |  |  |  |  |  |  |  |
| 3.16.6 | DPT |  |  |  |  |  |  |  |
| 3.16.7 | Measles |  |  |  |  |  |  |  |
| 3.16.8 | MR |  |  |  |  |  |  |  |
| 3.16.9 | TT |  |  |  |  |  |  |  |
| 3.16.10 | IPV |  |  |  |  |  |  |  |
| 3.16.11 | PCV |  |  |  |  |  |  |  |
| 3.16.12 | JE |  |  |  |  |  |  |  |
| 3.16.13 | MMR |  |  |  |  |  |  |  |

3.17 Month: **May** **2017**

| Q no | Vaccine | Start balance  (A) | No of doses received  (B) | No of doses discarded (unopened) (C) | No of doses issued (D) | No of doses returned (E) | No of doses vaccinated (children/  pregnant women) (F) | End balance  G= (A+B)-(C+D-E) |
| --- | --- | --- | --- | --- | --- | --- | --- | --- |
| 3.17.1 | BCG |  |  |  |  |  |  |  |
| 3.17.2 | OPV |  |  |  |  |  |  |  |
| 3.17.3 | HBV |  |  |  |  |  |  |  |
| 3.17.4 | Penta |  |  |  |  |  |  |  |
| 3.17.5 | Rotavirus |  |  |  |  |  |  |  |
| 3.17.6 | DPT |  |  |  |  |  |  |  |
| 3.17.7 | Measles |  |  |  |  |  |  |  |
| 3.17.8 | MR |  |  |  |  |  |  |  |
| 3.17.9 | TT |  |  |  |  |  |  |  |
| 3.17.10 | IPV |  |  |  |  |  |  |  |
| 3.17.11 | PCV |  |  |  |  |  |  |  |
| 3.17.12 | JE |  |  |  |  |  |  |  |
| 3.17.13 | MMR |  |  |  |  |  |  |  |

3.18 Month: **June 2017**

| Q no | Vaccine | Start balance  (A) | No of doses received  (B) | No of doses discarded (unopened) (C) | No of doses issued (D) | No of doses returned (E) | No of doses vaccinated (children/  pregnant women) (F) | End balance  G= (A+B)-(C+D-E) |
| --- | --- | --- | --- | --- | --- | --- | --- | --- |
| 3.18.1 | BCG |  |  |  |  |  |  |  |
| 3.18.2 | OPV |  |  |  |  |  |  |  |
| 3.18.3 | HBV |  |  |  |  |  |  |  |
| 3.18.4 | Penta |  |  |  |  |  |  |  |
| 3.18.5 | Rotavirus |  |  |  |  |  |  |  |
| 3.18.6 | DPT |  |  |  |  |  |  |  |
| 3.18.7 | Measles |  |  |  |  |  |  |  |
| 3.18.8 | MR |  |  |  |  |  |  |  |
| 3.18.9 | TT |  |  |  |  |  |  |  |
| 3.18.10 | IPV |  |  |  |  |  |  |  |
| 3.18.11 | PCV |  |  |  |  |  |  |  |
| 3.18.12 | JE |  |  |  |  |  |  |  |
| 3.18.13 | MMR |  |  |  |  |  |  |  |

3.19 Month: **July 2017**

| Q no | Vaccine | Start balance  (A) | No of doses received  (B) | No of doses discarded (unopened) (C) | No of doses issued (D) | No of doses returned (E) | No of doses vaccinated (children/  pregnant women) (F) | End balance  G= (A+B)-(C+D-E) |
| --- | --- | --- | --- | --- | --- | --- | --- | --- |
| 3.19.1 | BCG |  |  |  |  |  |  |  |
| 3.19.2 | OPV |  |  |  |  |  |  |  |
| 3.19.3 | HBV |  |  |  |  |  |  |  |
| 3.19.4 | Penta |  |  |  |  |  |  |  |
| 3.19.5 | Rotavirus |  |  |  |  |  |  |  |
| 3.19.6 | DPT |  |  |  |  |  |  |  |
| 3.19.7 | Measles |  |  |  |  |  |  |  |
| 3.19.8 | MR |  |  |  |  |  |  |  |
| 3.19.9 | TT |  |  |  |  |  |  |  |
| 3.19.10 | IPV |  |  |  |  |  |  |  |
| 3.19.11 | PCV |  |  |  |  |  |  |  |
| 3.19.12 | JE |  |  |  |  |  |  |  |
| 3.19.13 | MMR |  |  |  |  |  |  |  |

3.20 Month: **August 2017**

| Q no | Vaccine | Start balance  (A) | No of doses received  (B) | No of doses discarded (unopened) (C) | No of doses issued (D) | No of doses returned (E) | No of doses vaccinated (children/  pregnant women) (F) | End balance  G= (A+B)-(C+D-E) |
| --- | --- | --- | --- | --- | --- | --- | --- | --- |
| 3.20.1 | BCG |  |  |  |  |  |  |  |
| 3.20.2 | OPV |  |  |  |  |  |  |  |
| 3.20.3 | HBV |  |  |  |  |  |  |  |
| 3.20.4 | Penta |  |  |  |  |  |  |  |
| 3.20.5 | Rotavirus |  |  |  |  |  |  |  |
| 3.20.6 | DPT |  |  |  |  |  |  |  |
| 3.20.7 | Measles |  |  |  |  |  |  |  |
| 3.20.8 | MR |  |  |  |  |  |  |  |
| 3.20.9 | TT |  |  |  |  |  |  |  |
| 3.20.10 | IPV |  |  |  |  |  |  |  |
| 3.20.11 | PCV |  |  |  |  |  |  |  |
| 3.20.12 | JE |  |  |  |  |  |  |  |
| 3.20.13 | MMR |  |  |  |  |  |  |  |

3.21 Month: **September 2017**

| Q no | Vaccine | Start balance  (A) | No of doses received  (B) | No of doses discarded (unopened) (C) | No of doses issued (D) | No of doses returned (E) | No of doses vaccinated (children/  pregnant women) (F) | End balance  G= (A+B)-(C+D-E) |
| --- | --- | --- | --- | --- | --- | --- | --- | --- |
| 3.21.1 | BCG |  |  |  |  |  |  |  |
| 3.21.2 | OPV |  |  |  |  |  |  |  |
| 3.21.3 | HBV |  |  |  |  |  |  |  |
| 3.21.4 | Penta |  |  |  |  |  |  |  |
| 3.21.5 | Rotavirus |  |  |  |  |  |  |  |
| 3.21.6 | DPT |  |  |  |  |  |  |  |
| 3.21.7 | Measles |  |  |  |  |  |  |  |
| 3.21.8 | MR |  |  |  |  |  |  |  |
| 3.21.9 | TT |  |  |  |  |  |  |  |
| 3.21.10 | IPV |  |  |  |  |  |  |  |
| 3.21.11 | PCV |  |  |  |  |  |  |  |
| 3.21.12 | JE |  |  |  |  |  |  |  |
| 3.21.13 | MMR |  |  |  |  |  |  |  |

3.22 Month: **October** **2017**

| Q no | Vaccine | Start balance  (A) | No of doses received  (B) | No of doses discarded (unopened) (C) | No of doses issued (D) | No of doses returned (E) | No of doses vaccinated (children/  pregnant women) (F) | End balance  G= (A+B)-(C+D-E) |
| --- | --- | --- | --- | --- | --- | --- | --- | --- |
| 3.22.1 | BCG |  |  |  |  |  |  |  |
| 3.22.2 | OPV |  |  |  |  |  |  |  |
| 3.22.3 | HBV |  |  |  |  |  |  |  |
| 3.22.4 | Penta |  |  |  |  |  |  |  |
| 3.22.5 | Rotavirus |  |  |  |  |  |  |  |
| 3.22.6 | DPT |  |  |  |  |  |  |  |
| 3.22.7 | Measles |  |  |  |  |  |  |  |
| 3.22.8 | MR |  |  |  |  |  |  |  |
| 3.22.9 | TT |  |  |  |  |  |  |  |
| 3.22.10 | IPV |  |  |  |  |  |  |  |
| 3.22.11 | PCV |  |  |  |  |  |  |  |
| 3.22.12 | JE |  |  |  |  |  |  |  |
| 3.22.13 | MMR |  |  |  |  |  |  |  |

3.23 Month: **November** **2017**

| Q no | Vaccine | Start balance  (A) | No of doses received  (B) | No of doses discarded (unopened) (C) | No of doses issued (D) | No of doses returned (E) | No of doses vaccinated (children/  pregnant women) (F) | End balance  G= (A+B)-(C+D-E) |
| --- | --- | --- | --- | --- | --- | --- | --- | --- |
| 3.23.1 | BCG |  |  |  |  |  |  |  |
| 3.23.2 | OPV |  |  |  |  |  |  |  |
| 3.23.3 | HBV |  |  |  |  |  |  |  |
| 3.23.4 | Penta |  |  |  |  |  |  |  |
| 3.23.5 | Rotavirus |  |  |  |  |  |  |  |
| 3.23.6 | DPT |  |  |  |  |  |  |  |
| 3.23.7 | Measles |  |  |  |  |  |  |  |
| 3.23.8 | MR |  |  |  |  |  |  |  |
| 3.23.9 | TT |  |  |  |  |  |  |  |
| 3.23.10 | IPV |  |  |  |  |  |  |  |
| 3.23.11 | PCV |  |  |  |  |  |  |  |
| 3.23.12 | JE |  |  |  |  |  |  |  |
| 3.23.13 | MMR |  |  |  |  |  |  |  |

3.24 Month: **December** **2017**

| Q no | Vaccine | Start balance  (A) | No of doses received  (B) | No of doses discarded (unopened) (C) | No of doses issued (D) | No of doses returned (E) | No of doses vaccinated (children/  pregnant women) (F) | End balance  G= (A+B)-(C+D-E) |
| --- | --- | --- | --- | --- | --- | --- | --- | --- |
| 3.24.1 | BCG |  |  |  |  |  |  |  |
| 3.24.2 | OPV |  |  |  |  |  |  |  |
| 3.24.3 | HBV |  |  |  |  |  |  |  |
| 3.24.4 | Penta |  |  |  |  |  |  |  |
| 3.24.5 | Rotavirus |  |  |  |  |  |  |  |
| 3.24.6 | DPT |  |  |  |  |  |  |  |
| 3.24.7 | Measles |  |  |  |  |  |  |  |
| 3.24.8 | MR |  |  |  |  |  |  |  |
| 3.24.9 | TT |  |  |  |  |  |  |  |
| 3.24.10 | IPV |  |  |  |  |  |  |  |
| 3.24.11 | PCV |  |  |  |  |  |  |  |
| 3.24.12 | JE |  |  |  |  |  |  |  |
| 3.24.13 | MMR |  |  |  |  |  |  |  |

**4. Any specific comment/ observation.**

**Thank the Store in-charge/ cold chain handler his/her support.**
