# Supplementary material for: Documentation of vaccine wastage in two different geographic contexts under the universal immunization program in India
Source: BMC Public Health. 2020 Apr 25;20:556. doi: 10.1186/s12889-020-08637-1 (PMC7183620; doi:10.1186/s12889-020-08637-1)
Supplement: Supplementary file 3 — Additional file 3: Supplementary file 3. In-depth interview guides for stakeholders. Supplementary file 3.1: In-depth interview guide- Cold chain handler. Supplementary file 3.2: In-depth interview guide- Vaccinator . Supplementary file 3.3: In-depth interview guide- Store in-charge. Supplementary file 3.4: In-depth interview guide-Medical Officer. Supplementary file 3.5: In-depth interview guide-District Immunization Officer [file 12889_2020_8637_MOESM3_ESM.docx]

| **UNIQUE ID** |  |
| --- | --- |

**Documentation of vaccine wastage under the universal immunization program in the districts of Kangra (Himachal Pradesh) and Pune (Maharashtra)**

**In-depth Interview**

**Cold Chain Handler (PHC/CHC/DH)**

| **District Name** | **Kangra Pune** |
| --- | --- |
| **Date of Interview** |  |
| **Start time** | (HH: MM) |
| **End time** | (HH: MM) |

1. **General Questions**

Please obtain the following information for the stakeholder whom you are meeting for interview.

| 1.1 | **Designation** |  |
| --- | --- | --- |
| 1.2 | **Period in present post** (years & months) |  |
| 1.3 | **Total years in service** |  |

1. **What all and how many cold chain equipment are available at your facility?**

1. **How do you undertake maintenance care of the cold chain equipment?**

(Types of maintenance practices and frequency)

(Practices like defrosting, temperature setting, ice cleaning from wall, etc.….)

1. **How power supply for the cold chain equipment is ensured?**

(ask for ensuring continued power supply, handling power cuts)

1. **Are separate thermometers for measuring temperature of the ILR and DF available for all the equipment?**
2. **How do you monitor the temperature for the ILR and DF and at what frequency?**
3. **What all do your do to prevent freezing of the vaccines, especially the freezing-rick vaccines (pentavalent, DPT, TT, HBV)?**

(Ask for storage place, preventing touching the wall of ILR)

1. **How do you store the opened vials from the session sites?**
2. **How do you handle the used and empty vaccine vials received from the session site?**
3. **Have you encountered any vaccine crossed expiry date or VVM unusable stage?**

If yes, how did you handle the situation? If yes, what was the possible reason?

1. **Have you ever encountered freezing of freeze-sensitive vaccines?**

If yes, how did you handle the situation?

If yes, what was the possible reason?

1. **What do you do to avoid expiry of the vaccine or reaching VVM unusable stage?**
2. **How and at what frequency the reporting is done to the higher level?**
3. **How vaccine wastage at your facility and in the area is being tracked and monitored?**
4. **Have you ever encountered vaccine wastage due to freezing or damaged vials at your facility?**

(Who does it, how and at what frequency?)

1. **What are the challenges faced by you in the process of cold chain handling and vaccine handling?**
2. **Who provides the supervision and monitoring support for the cold chain and vaccine handling?**
3. **Do you get any feedback on the immunization and vaccine related report submitted?**

If yes, please give an example.

1. **What changes in vaccine utilization did you/your team experience with change from 5-doses Rotavirus vaccine vial to 10-doses vial?**
2. **What changes in vaccine utilization did you/your team experience with change from IPV IM doses to f-IPV?**
3. **Any other suggestion or feedback.**

**Thank him/her for the inputs and cooperation.**

| **UNIQUE ID** |  |
| --- | --- |

**Documentation of vaccine wastage under the universal immunization program in the districts of Kangra (Himachal Pradesh) and Pune (Maharashtra)**

**In-depth Interview**

**Vaccinator (LHV/ANM/Nurse)**

| **District Name** | **Kangra Pune** |
| --- | --- |
| **Date of Interview** |  |
| **Start time** | (HH: MM) |
| **End time** | (HH: MM) |

1. **General Questions**

Please obtain the following information for the stakeholder whom you are meeting for interview.

| 1.1 | **Designation** |  |
| --- | --- | --- |
| 1.2 | **Period in present post** (years & months) |  |
| 1.3 | **Total years in service** |  |

1. **Please let us know about the immunization session you conduct?**

(Type of session and place- facility/ sub-center/outreach)

1. **How do you estimate the vaccine requirement for the sessions?**

(Frequency of estimation and indenting/requesting)

1. **Who prepares the due list for the scheduled sessions and how do you receive the list, if you don’t prepare?**
2. **How do you usually receive the vaccines (vaccine carrier) at the session site?**

(Mode of the transport used and person who carries)

1. **Do you receive any challan/ issue sheet along with the vaccine carrier? If received, what all are usually mentioned in the sheet?**

(vaccines, batch number, expiry number, VVM status, open or unopened vialm number of remaining doses in opened vial)

1. **How are the vaccines placed inside the carrier when you receive them at the session?**

(Number of ice packs used, placement of the vaccines and care for preventing direct contact with the ice packs)

1. **Have you ever experienced freezing of any vaccine vial when received at the session site?**
2. **How frequently do you conduct freeze test and for which vaccines?**
3. **How do you record the doses administered during the session?**

(writing in the due list or in tally sheet or separate sheet)

1. **How do you handle the used and empty vaccine vials after the session?**
2. **Have you experienced any vaccine stock out situation in last one year? If yes, how did you handle the situation?**
3. **Do you send session performance report, at what frequency and to whom?**
4. **How do you enter the vaccination performance reports in the RCH register and at what frequency?**
5. **What are the challenges faced by you in organizing and conducting the sessions?**
6. **Who provides the supervision and monitoring support for your sessions?**
7. **Do you get any feedback on the immunization and vaccine related report submitted?**

**If yes, please give an example.**

1. **What changes in vaccine utilization and wastage did you/your team experience with change from 5-doses Rotavirus vaccine vial to 10-doses vial?**
2. **What changes in vaccine utilization and wastage did you/your team experience with change from IPV IM doses to f-IPV?**
3. **Any other suggestion or feedback.**

**Thank him/her for the inputs and cooperation.**

| **UNIQUE ID** |  |
| --- | --- |

**Documentation of vaccine wastage under the universal immunization program in the districts of Kangra (Himachal Pradesh) and Pune (Maharashtra)**

**In-depth Interview**

**Store in-charge (PHC/CHC/DH)**

| **District Name** | **Kangra Pune** |
| --- | --- |
| **Date of Interview** |  |
| **Start time** | (HH: MM) |
| **End time** | (HH: MM) |

1. **General Questions**

Please obtain the following information for the stakeholder whom you are meeting for interview.

| 1.1 | **Designation** |  |
| --- | --- | --- |
| 1.2 | **Period in present post** (years & months) |  |
| 1.3 | **Total years in service** |  |

1. **How is estimation of vaccine and related supplies for this facility done?**

(who prepares, method of estimation, frequency and inputs used)

1. **How is the indenting for vaccines and related supplies done for this facility?**

(person responsible, frequency and mode of sending)

1. **How do you receive the vaccines and related supplies for this facility?**

(collected or supplied and frequency of receipt)

1. **How do you record the vaccines received, in stock and used vaccines?**

(Types of maintenance practices and frequency)

1. **Have you experienced any vaccine wastage of damage at this facility/store?**

**If yes, how was the vaccine wastage documented in the stock register?**

(ask for vaccine vial damage, VVM reaching unusable stage or crossing expiry date)

1. **How frequently physical stock check for vaccines is done and by whom?**
2. **How and at what frequency the reporting is done to the higher level?**
3. **How vaccine wastage at your facility and in the area is being tracked and monitored?**
4. **What are the challenges faced by you related to vaccine stock keeping?**
5. **Who provides the supervision and monitoring support for the stock keeping?**
6. **What changes in vaccine utilization did you/your team experience with change from 5-doses Rotavirus vaccine vial to 10-doses vial?**
7. **What changes in vaccine utilization did you/your team experience with change from IPV IM doses to f-IPV?**
8. **Any other suggestion or feedback.**

**Thank him/her for the inputs and cooperation.**

| **UNIQUE ID** |  |
| --- | --- |

**Documentation of vaccine wastage under the universal immunization program in the districts of Kangra (Himachal Pradesh) and Pune (Maharashtra)**

**In-depth Interview**

**Medical Officer in-charge (PHC/CHC)**

| **District Name** | **Kangra Pune** |
| --- | --- |
| **Date of Interview** |  |
| **Start time** | (HH: MM) |
| **End time** | (HH: MM) |

1. **General Questions**

Please obtain the following information for the stakeholder whom you are meeting for interview.

| 1.1 | **Designation** |  |
| --- | --- | --- |
| 1.2 | **Period in present post** (years & months) |  |
| 1.3 | **Total years in service** |  |

1. **How is estimation of vaccine and related supplies for this facility and the area covered under this facility done?**

(who prepares, method of estimation, frequency and inputs used)

1. **How is the indenting for vaccines and related supplies done for this facility?**

(person responsible, frequency and mode of sending)

1. **How does the facility receive the vaccines and related supplies?**

(collected or supplied and frequency of receipt)

1. **How the vaccine storage and temperature maintenance practices are supervised at the facility?**

(who does it, frequency and documentation)

1. **How frequently physical stock check for vaccines is done and by whom?**
2. **Have you experienced any vaccine wastage of damage at this facility/store?**

**If yes, how was the vaccine wastage documented in the stock register?**

(ask for vaccine vial damage, VVM reaching unusable stage or crossing expiry date)

1. **What steps are taken at the facility level to avoid vaccine wastage?**
2. **How vaccine wastage at your facility and in the area is being tracked and monitored?**
3. **How and at what frequency the reporting is done to the higher level?**
4. **What are the challenges faced by you related to vaccine stock keeping?**
5. **Who provides the supervision and monitoring support for the vaccine handling, stock keeping?**
6. **What changes in vaccine utilization and wastage did you/your team experience with change from 5-doses Rotavirus vaccine vial to 10-doses vial?**
7. **Did you make any adjustment/modification in your program implementation activities for the same?**
8. **What changes in vaccine utilization and wastage did you/your team experience with change from IPV IM doses to f-IPV?**
9. **Did you make any adjustment/modification in your program implementation activities for the same?**
10. **Any other suggestion or feedback.**

**Thank him/her for the inputs and cooperation.**

| **UNIQUE ID** |  |
| --- | --- |

**Documentation of vaccine wastage under the universal immunization program in the districts of Kangra (Himachal Pradesh) and Pune (Maharashtra)**

**In-depth Interview**

**District Immunization Officer**

| **District Name** | **Kangra Pune** |
| --- | --- |
| **Date of Interview** |  |
| **Start time** | (HH: MM) |
| **End time** | (HH: MM) |

1. **General Questions**

Please obtain the following information for the stakeholder whom you are meeting for interview.

| 1.1 | **Designation** |  |
| --- | --- | --- |
| 1.2 | **Period in present post** (years & months) |  |
| 1.3 | **Total years in service** |  |

1. **How is estimation of vaccine and related supplies for this facility and the area covered under this facility done?**

(who prepares, method of estimation, frequency and inputs used)

1. **How is the indenting for vaccines and related supplies done for this district?**

(person responsible, frequency and mode of sending)

1. **How are the vaccine and related supplies supplied to various facilities/stores in the district?**

(collected or supplied and frequency of receipt)

1. **How the vaccine storage and temperature maintenance practices are supervised in the district?**

(who does it, frequency and documentation)

1. **How frequently physical stock check for vaccines is done and by whom?**
2. **Have you experienced any vaccine wastage of damage at this facility/store?**

**If yes, how was the situation handled?**

(ask for vaccine vial damage, VVM reaching unusable stage or crossing expiry date)

1. **What steps are taken in the district to avoid vaccine wastage?**
2. **How vaccine wastage in the district is being tracked and monitored?**
3. **How and at what frequency the reporting is done to the state level?**
4. **What are the challenges faced by you related to vaccine logistics and cold chain maintenance?**
5. **Who provides the supervision and monitoring support for the vaccine handling, stock keeping?**
6. **What changes in vaccine utilization and wastage did you/your team experience with change from 5-doses Rotavirus vaccine vial to 10-doses vial?**
7. **Did you make any adjustment/modification in your program implementation activities for the same?**
8. **What changes in vaccine utilization and wastage did you/your team experience with change from IPV IM doses to f-IPV?**
9. **Did you make any adjustment/modification in your program implementation activities for the same?**
10. **Any other suggestion or feedback.**

**Thank him/her for the inputs and cooperation.**
