# Supplementary material for: Documentation of vaccine wastage in two different geographic contexts under the universal immunization program in India
Source: BMC Public Health. 2020 Apr 25;20:556. doi: 10.1186/s12889-020-08637-1 (PMC7183620; doi:10.1186/s12889-020-08637-1)
Supplement: Supplementary file 4 — Additional file 4: Table S1. Wastage rates (as percentage) according to the type of sessions [file 12889_2020_8637_MOESM4_ESM.docx]

**Supplementary file 4**

Supplementary Table S1: Wastage rates (as percentage) according to the type of sessions

|  | Level | BCG | DPT | HBV | IPV | Measels | OPV | LPV | RVV | TT | MR | PCV |
| --- | --- | --- | --- | --- | --- | --- | --- | --- | --- | --- | --- | --- |
| Kangra | SDH/  RH/  CHC | 35.4 | 29.3 | 30.3 | 48.9 | 31.6 | 48.1 | 17.8 | 31.7 | 31.9 | 42.4 | 30.1 |
|  | PHC | 54.1 | 41.5 | 25 | 57.4 | 37.2 | 59.3 | 23.4 | 33.2 | 46.7 | 19.8 | 22.0 |
|  | OR | 32.6 | - | - | - | 21.8 | - | - | 29.7 |  | 4.8 | - |
| Pune | SDH/  RH/  CHC | 23.4 | 23.5 | 10.8 | 18.1 | 11.6 | 4.4 | 10.4 | 20.0 | 26.5 | - | - |
|  | PHC | 37.5 | 25.5 | 22.4 | 27.6 | 22.3 | 15.3 | 13.5 | 23.6 | 22.7 | - | - |
|  | OR | 24.9 | - | - | - | 27.4 | - | - | 20.1 |  | - | - |

*Note: SDH: Subdivision hospital; RH: Rural hospital; CHC: Community health centre; PHC: Primary health centre; OR: Outreach sessions; OR: Outreach sessions (includes the sessions at sub-centres and outreach sites)*

*DPT: Diptheria-pertusis-tetanus; HBV: Hepatitis B vaccine; IPV: Inactivated polio vaccine; OPV: Oral polio vaccine; LPV: Liquid pentavalent vaccine; RVV: Rotavirus vaccine; TT: Tetanus toxoid; MR: Measles and rubella; and PCV: Pneumococcal conjugate vaccine.*
