# Supplementary material for: Documentation of vaccine wastage in two different geographic contexts under the universal immunization program in India
Source: BMC Public Health. 2020 Apr 25;20:556. doi: 10.1186/s12889-020-08637-1 (PMC7183620; doi:10.1186/s12889-020-08637-1)
Supplement: Supplementary file 5 — Additional file 5: Table S2. The trend of vaccine wastage across months in Kangra district (pooled for the district) [file 12889_2020_8637_MOESM5_ESM.docx]

**Supplementary file 5: Supplementary Table**

Supplementary Table S2: The trend of vaccine wastage across months in Kangra district (pooled for the district)

|  |  |  |  |  |  |  |  |  |  |  |  |
| --- | --- | --- | --- | --- | --- | --- | --- | --- | --- | --- | --- |
| Month | BCG | OPV | DPT | HBV | LPV | IPV | Measles | MR | PCV | RVV | TT |
| Jan-16 | 39% | 38% | 33% | 29% | 22% | - | 29% | - | - | 29% | 3% |
| Feb-16 | 39% | 42% | 28% | 32% | 19% | - | 32% | - | - | 22% | 28% |
| Mar-16 | 44% | 23% | 34% | 38% | 28% | - | 32% | - | - | 21% | 27% |
| Apr-16 | 38% | 42% | 34% | 2% | 21% | 27% | 39% | - | - | 22% | 33% |
| May-16 | 29% | 50% | 32% | 36% | 15% | 31% | 26% | - | - | 26% | 18% |
| Jun-16 | 43% | 29% | 24% | 51% | 15% | 48% | 26% | - | - | 25% | 39% |
| Jul-16 | 38% | 28% | 27% | 36% | 14% | 38% | 24% | - | - | 21% | 31% |
| Aug-16 | 35% | 34% | 28% | 45% | 11% | 35% | 30% | - | - | 26% | 28% |
| Sep-16 | 34% | 27% | 22% | 11% | 16% | 32% | 23% | - | - | 34% | 7% |
| Oct-16 | 41% | 25% | 27% | 41% | 12% | 32% | 34% | - | - | 29% | 52% |
| Nov-16 | 33% | 36% | 29% | 9% | 11% | 28% | 28% | - | - | 16% | 27% |
| Dec-16 | 35% | 23% | 35% | 24% | 16% | 35% | 21% | - | - | 23% | 25% |
| Jan-17 | 31% | 38% | 30% | 13% | 16% | 36% | 30% | - | - | 32% | 31% |
| Feb-17 | 34% | 25% | 24% | 27% | 18% | 38% | 29% | - | - | 27% | 23% |
| Mar-17 | 30% | 30% | 43% | 42% | 19% | 40% | 32% | - | - | 37% | 25% |
| Apr-17 | 26% | 30% | 28% | 52% | 14% | 42% | 32% | - | - | 34% | 29% |
| May-17 | 37% | 43% | 32% | 48% | 18% | 49% | 34% | - | - | 29% | 48% |
| Jun-17 | 34% | 30% | 28% | 43% | 22% | 36% | 23% | - | 27% | 37% | 17% |
| Jul-17 | 40% | 35% | 24% | 48% | 22% | 43% | 28% | - | 30% | 44% | 41% |
| Aug-17 | 40% | 26% | 35% | 44% | 23% | 38% | 33% | 78% | 12% | 39% | 18% |
| Sep-17 | 42% | 37% | 34% | 18% | 26% | 44% | 8% | 57% | 14% | 29% | 50% |
| Oct-17 | 26% | 31% | 31% | 33% | 11% | 50% | 8% | 66% | 14% | 33% | 46% |
| Nov-17 | 37% | 29% | 37% | 8% | 22% | 50% | - | 68% | 33% | 36% | 36% |
| Dec-17 | 42% | 28% | 27% | 21% | 20% | 41% | - | 69% | 24% | 34% | 29% |

*Note: DPT: Diptheria-pertusis-tetanus; HBV: Hepatitis B vaccine; IPV: Inactivated polio vaccine; OPV: Oral polio vaccine; LPV: Liquid pentavalent vaccine; RVV: Rotavirus vaccine; TT: Tetanus toxoid; MR: Measles and rubella; and PCV: Pneumococcal conjugate vaccine.*
