# Supplementary material for: Documentation of vaccine wastage in two different geographic contexts under the universal immunization program in India
Source: BMC Public Health. 2020 Apr 25;20:556. doi: 10.1186/s12889-020-08637-1 (PMC7183620; doi:10.1186/s12889-020-08637-1)
Supplement: Supplementary file 6 — Additional file 6: Table S3. The trend of vaccine wastage across months in Pune district (pooled for the district) [file 12889_2020_8637_MOESM6_ESM.docx]

**Supplementary file 6: Supplementary Table**

Supplementary Table S3: The trend of vaccine wastage across months in Pune district (pooled for the district)

| Pune |  |  |  |  |  |  |  |  |  |
| --- | --- | --- | --- | --- | --- | --- | --- | --- | --- |
| Month | BCG | OPV | DPT | HBV | LPV | IPV | Measles | RVV | TT |
| Jan-16 | 47% | 21% | 32% | 28% | 27% |  | 25% | 36% | 29% |
| Feb-16 | 42% | 28% | 19% | 34% | 25% |  | 27% | 33% | 22% |
| Mar-16 | 51% | 26% | 23% | 53% | 23% |  | 29% | 31% | 35% |
| Apr-16 | 46% | 23% | 37% | 37% | 28% | 50% | 37% | 28% | 23% |
| May-16 | 38% | 24% | 41% | 47% | 20% | 14% | 23% | 26% | 47% |
| Jun-16 | 37% | 20% | 28% | 25% | 19% | 17% | 29% | 24% | 45% |
| Jul-16 | 31% | 20% | 27% | 22% | 16% | 20% | 34% | 19% | 33% |
| Aug-16 | 42% | 24% | 23% | 14% | 19% | 20% | 32% | 17% | 33% |
| Sep-16 | 42% | 24% | 28% | 19% | 17% | 20% | 26% | 26% | 29% |
| Oct-16 | 41% | 25% | 37% | 24% | 10% | 30% | 26% | 31% | 38% |
| Nov-16 | 36% | 26% | 29% | 20% | 14% | 20% | 24% | 28% | 33% |
| Dec-16 | 42% | 29% | 42% | 34% | 21% | 20% | 30% | 29% | 38% |
| Jan-17 | 32% | 21% | 40% | 40% | 29% | 37% | 38% | 36% | 26% |
| Feb-17 | 39% | 16% | 22% | 29% | 23% | 49% | 27% | 32% | 49% |
| Mar-17 | 37% | 29% | 42% | 16% | 23% | 0% | 29% | 38% | 33% |
| Apr-17 | 43% | 17% | 39% | 24% | 26% | 18% | 29% | 39% | 40% |
| May-17 | 35% | 22% | 37% | 25% | 22% | 41% | 33% | 35% | 23% |
| Jun-17 | 41% | 21% | 39% | 28% | 22% | 37% | 37% | 36% | 15% |
| Jul-17 | 31% | 25% | 33% | 25% | 25% | 21% | 33% | 33% | 37% |
| Aug-17 | 37% | 22% | 23% | 8% | 23% | 24% | 37% | 39% | 30% |
| Sep-17 | 21% | 10% | 15% | 18% | 26% | 30% | 34% | 33% | 20% |
| Oct-17 | 26% | 26% | 18% | 26% | 15% | 36% | 30% | 27% | 23% |
| Nov-17 | 29% | 18% | 33% | 23% | 14% | 17% | 27% | 28% | 12% |
| Dec-17 | 44% | 15% | 31% | 26% | 19% | 13% | 23% | 29% | 10% |

*Note: OPV: Oral polio vaccine; DPT: Diptheria-pertusis-tetanus; HBV: Hepatitis B vaccine; IPV: Inactivated polio vaccine; LPV: Liquid pentavalent vaccine; RVV: Rotavirus vaccine; and TT: Tetanus toxoid*
