# Supplementary material for: Documentation of vaccine wastage in two different geographic contexts under the universal immunization program in India
Source: BMC Public Health. 2020 Apr 25;20:556. doi: 10.1186/s12889-020-08637-1 (PMC7183620; doi:10.1186/s12889-020-08637-1)
Supplement: Supplementary file 7 — Additional file 7: Supplementary file 7. Statements of stakeholders regarding the vaccine wastage and strategies to minimise wastage [file 12889_2020_8637_MOESM7_ESM.docx]

**Additional File 2: Statements of stakeholders regarding the vaccine wastage and strategies to minimise wastage**

“With five dose rotavirus vaccine vial wastage was less. Session load is usually for 3-5 doses. With the ten dose vials, wastage is about 5-7 doses. To minimise, the vaccinators call the children sometimes to next nearby session. These changes are done by the vaccinators at their own level.” *(District Immunization Officer, Kangra 1101112)*

“Wastage of vaccine is there, but they rearrange the immunization sessions to avoid it.”

*(Store in-charge, Kangra 1109313)*

“Routine immunization days reduced and being held together with nearby areas.”

*(Store in-charge, Kangra 1107313)*

“When ten dose vial came, BMOs/MOs/HWs were asked to reschedule the session so that the wastage is minimised.” *(Cold chain handler, Kangra 1101114)*

“There is more wastage with ten dose vials. We have reduced the number of sessions.” *(Medical Officer, Kangra 1101152)*

“If less number of beneficiaries are there, they are referred to nearby sub-centre and PHC.” *(Medical Officer, Kangra 1106252)*

“In order to prevent wastage, five dose vials are required or open vial policy should be implemented.” *(Vaccinator, Kangra 1112416)*

“Vaccine wastage was less with five doses. We have made no major adjustment.”

*(District Immunization Officer, Pune 2100112)*

“They try to club children, so that the vaccine wastage can be minimised.”

*(Store in-charge, Pune 2106533)*

“ANM is advised to plan the session according to the due list and are sked to club the beneficiaries, so that the wastage can be minimal.” *(Store in-charge, Pune 2103223)*

“Try to club the beneficiaries, so that the wastage can be reduced.”

*(Store in-charge, Pune 2104233)*

“The wastage rate is more at sub-centre and outreach sessions with ten dose vial. At PHC ten dose vial can be used.” *(Cold chain handler, Pune 2102214)*

“Ten dose vial is better, because changing to new vials take more time for the session if five dose vial is used.” *(Cold chain handler, Pune 2102224)*

“We have to wait till more beneficiaries gather when ten dose vial is to be opened. But even if single beneficiary is there, we have to open the ten dose vial after waiting, this has increased the wastage.” *(Cold chain handler, Pune 2106524)*

“For sub-centres, only if enough beneficiaries are there, then they open the 10 dose vial. They try to gather beneficiaries before the session.” *(Medical Officer, Pune 2104232)*

“We felt that 10 dose vial is better, as many times when session has more number of beneficiaries, more 5 dose vials are needed. So there is space problem in the vaccine carrier. When we talk about wastage, 10 dose has more, but it doesn’t make much difference, because many times when more than five beneficiaries are there, we have to open another 5 dose vial.” *(Vaccinator, Pune 2103416)*
